# Embodied science and mixed reality: How gesture and motion capture affect physics education

**DOI:** 10.1186/s41235-017-0060-9

**Published:** 2017-05-24

**Authors:** Mina C. Johnson-Glenberg, Colleen Megowan-Romanowicz

**Affiliations:** 1 Department of Psychology, Arizona State University, Tempe, AZ USA; 2 Embodied Games LLC, Tempe, AZ USA; 3 Modeling Instruction Institute, Sacramento, CA USA

**Keywords:** Virtual reality, Mixed reality, Embodied science, Science education, Physics, STEM, Game-based learning, Narrative, Gesture and learning

## Abstract

A mixed design was created using text and game-like multimedia to instruct in the content of physics. The study assessed which variables predicted learning gains after a 1-h lesson on the electric field. The three manipulated variables were: (1) level of embodiment; (2) level of active generativity; and (3) presence of story narrative. Two types of tests were administered: (1) a traditional text-based physics test answered with a keyboard; and (2) a more embodied, transfer test using the *Wacom* large tablet where learners could use gestures (long swipes) to create vectors and answers. The 166 participants were randomly assigned to four conditions: (1) symbols and text; (2) low embodied; (3) high embodied/active; or (4) high embodied/active with narrative. The last two conditions were active because the on-screen content could be manipulated with gross body gestures gathered via the *Kinect* sensor. Results demonstrated that the three groups that included embodiment learned significantly more than the symbols and text group on the traditional keyboard post-test. When knowledge was assessed with the *Wacom* tablet format that facilitated gestures, the two active gesture-based groups scored significantly higher. In addition, engagement scores were significantly higher for the two active embodied groups. The *Wacom* results suggest test sensitivity issues; the more embodied test revealed greater gains in learning for the more embodied conditions. We recommend that as more embodied learning comes to the fore, more sensitive tests that incorporate gesture be used to accurately assess learning. The predicted differences in engagement and learning for the condition with the graphically rich story narrative were not supported. We hypothesize that a narrative effect for motivation and learning may be difficult to uncover in a lab experiment where participants are primarily motivated by course credit. Several design principles for mediated and embodied science education are proposed.

**Electronic supplementary material:**

The online version of this article (doi:10.1186/s41235-017-0060-9) contains supplementary material, which is available to authorized users.

## Significance

New and affordable motion tracking sensors are driving educational designers to consider including more gesture and body movements into lessons for the classroom. Principles of embodied cognition (Barsalou, 2008; Glenberg, 2008; Wilson, 2003) suggest that including movement and gesture is likely to benefit learning of even abstract information, such as concepts in mathematics (Alibali & Nathan, 2012) and physics (Kontra, Lyons, Fischer, & Beilock, 2015). Indeed, movement holds a special place for educational innovators, Maria Montessori wrote, “Movement, or physical activity, is thus an essential factor in intellectual growth, which depends upon the impressions received from outside. Through movement we come in contact with external reality, and it is through these contacts that we eventually acquire even abstract ideas” p.36 (Montessori, 1966).

The research reported in this article applies a taxonomy of levels of embodiment (Johnson-Glenberg, Birchfield, Koziupa, & Tolentino, 2014a; Johnson-Glenberg, Megowan-Romanowicz, Birchfield, & Savio-Ramos, 2016) to design four ways of teaching abstract concepts related to the electric field. In addition, we examine how different modes of testing (a more traditional keyboard-based assessment versus a gesture-based assessment) might further inform our understanding of embodied education. In the remainder of this introduction, we briefly review the notion of embodied cognition, the taxonomy of levels of embodiment, and the special role of gesture in learning. In addition, we consider the potential further impact of adding a game-like narrative to the teaching of electric fields. We end the introduction with the four research questions explored in the experiment.

## Mediated embodiment

Educational technology is moving rapidly and our theories and design principles need to keep pace. Some principles are beginning to emerge for embodiment in mixed reality (Lindgren & Johnson-Glenberg, 2013) and learning in augmented reality spaces (Dunleavy, 2014; Dunleavy & Dede, 2014). Nonetheless, as experimental psychologists and educational designers for mediated (computerized) content, we need to keep researching and striving to understand the optimal pedagogies for new media. As a lab, we have been creating science education content for years in embodied Mixed Reality (MR) platforms. MR means that elements of the tangible, physical world are mixed with virtual and digitized components (Milgram & Kishino, 1994). We have often relied on somewhat traditional tools to assess knowledge change, e.g. multiple choice pre- and post-tests using paper and pencil (Birchfield & Johnson-Glenberg, 2012; Johnson-Glenberg, Birchfield, Megowan-Romanowicz, Tolentino, & Martinez, 2009). Others have automated the process and made it moderately more embodied (Segal, Black, & Tversky, 2010). Recently, our group analyzed embodied motor behaviors in a *Kinect*-based learning environment and correlated process performance to the more traditional pre- and post-tests (Johnson-Glenberg, Birchfield, Megowan-Romanowicz, & Snow, 2015). We are trying to move towards more embodied and process-oriented methodologies for assessment. It is non-trivial to link learning and movement data because both the learning scenario and the movement tasks must be designed from the very beginning to yield meaningful and capturable constructs predicted to alter. That is, you must know the action you wish to capture, then design gesture instances into the learning activity, and measure the cognitive and behavioral change over meaningful time bins. The research in this article was driven by two overarching goals: there is the design goal to create optimal content; and there is the assessment goal to explore a testing format that will be sensitive to knowledge gathered when learners encode content via gesture.

Educational content is never simply embodied or not; there are most certainly degrees. Reading a text-only passage that is visually evocative is embodied, albeit we would consider that experience to be low embodied. If perceptual symbols are always activated (Barsalou, 1999) even during daydreaming, then it is problematic to state that some content evokes zero embodiment. Thus, we avoid terminology like “the condition with no embodiment.” Barsalou (1999) claims that abstract concepts are “…grounded in complex simulations of combined physical and introspective events.” The amount of embodiment experienced by a learner will therefore be nuanced and personalized. As a field, we need more methodical descriptors for the levels of embodiment in lessons.

## The taxonomy of embodiment in education

To that end, we proposed a taxonomy. The taxonomy with four degrees of embodiment for new media follows a “weak ordering” system (Johnson-Glenberg et al., 2014a, 2014b, 2016). The degrees depend on three constructs that are not strictly orthogonal. The degrees of embodiment are predicated on the constructs of:

- amount of sensorimotor engagement,
- how congruent the gestures are to the content to be learned, and
- amount of immersion experienced by the user.

This study varied the first two constructs; the final construct of immersion was held constant in that each condition viewed the same large projection area, a 78-inch diagonal. The first two constructs are not unrelated, because for a gesture to be congruent, there must be some amount of sensorimotor engagement. Nonetheless, within these constructs magnitudes can vary and these affect the overall degree. There are four conditions in the study. The decision to label the lessons as low or high embodied is guided by this taxonomy. The taxonomy breaks down the continuous spectrum of embodiment into four degrees with the fourth being the highest. The anchor points of the fourth degree—high in all constructs—and the first degree—low in all constructs—are well-justified, but there could be discussion regarding which constructs count as the most important for the third or second degrees. The taxonomy represents an improvement beyond the simplistic claim that educational content is either “embodied or not.” Table 1 highlights the four degrees by magnitude of construct. Below we describe the degrees in more detail.

**Table 1:** Construct magnitude within degrees in the Embodied Education Taxonomy

Degree	4th	3rd	3rd	3rd	2nd	2nd	2nd	1st
Embodiment construct								
Sensorimotor	H	H	H^a^	L	L	L	H^a^	L
Gestural congruency	H	H	L^a^	H	L	H	L^a^	L
Immersion	H	L	H	H	H	L	L	L

^a^This pairing could exist, but it would be ill-conceived to require a large movement that was poorly mapped to the content to be learned

*H* high, *L* low

*Fourth degree* = All three constructs need to be rated as being high. (1) Sensorimotor engagement: for gesture to be mapped to the lesson, some sort of sensor is used to link (e.g. via motion capture, etc.) the whole body, or multiple limbs, to the actionable components of the lesson. The body, or limbs, can act as the controller and the learner is able to manipulate what is happening on a display. If locomotion is included, then visual parallax is also engaged and this is an important signal (Campos et al., 2000), as it further increases sensorimotor activation. Multimodal effects (e.g. auditory and haptic cues) are present in fourth degree lessons and these increase sensorimotor activation. (2) Gestural congruency: within a lesson there are multiple instances of gestures that drive the system and those gestures are consistently designed to map to the content being learned, e.g. spinning the arm makes a virtual gear spin the same speed and same direction on the screen. This is congruent to and aids in the learning of the educational goal, in the gears example the learning goal might be mechanical advantage (Johnson-Glenberg et al., 2015). (3) Sense of immersion: immersion is related to the sense of being there. Slater and others have published extensively on immersion and presence (Slater, Spanlang, & Corominas, 2010; Slater & Wilbur, 1997). Slater considers immersion to be a property of the platform and presence is the construct that describes how much the learner “feels they are there.” Other theorists are comfortable with the term immersion also encompassing presence (Dede, Richards, & Jacobson, in press). In this article, immersion is a property of the platform which did not alter between all conditions. There are several methods, primarily survey, for measuring immersion, we use size of the display area. Display areas vary from smart phones screens to wrap-around 360° head-mounted displays (HMD) used in virtual reality (VR). We used a very large projection screen and borders were present in the periphery (borderless displays are generally considered more immersive).

*Third degree* = (1) Sensorimotor engagement: the whole body could be used as the controller, but the user remains in one place (e.g. standing at an interactive whiteboard). At least one large physical gesture (beyond finger movement) should be present and mapped to the content. (2) Gestural congruency: the system should contain one or more instances of a gesture that is well-mapped to the content. (3) Sense of immersion: a large screen display or floor projection should induce the learner to perceive the environment as immersive; however, borders are usually present in the peripheral field of vision (FOV).

*Second degree* = (1) Sensorimotor engagement: learner is generally seated, but there is some upper body movement of the arm or fingers. (2) Gestural congruency: this is probably not a defining construct in the lesson, although there is always some interactivity (e.g. a finger swipe to advance, or a flick-wrist-forward action while holding a smart phone to simulate casting a fishing reel). (3) Sense of immersion: the display covers less than 50% of the FOV; borders and real world are always present no matter the fixation point (e.g. a 16-inch monitor or tablet-sized screen).

*First degree* = (1) Sensorimotor engagement: learner is generally seated, but there is some upper body movement, but usually just for a key press. The learner is primarily observing a video/simulation. (2) Gestural congruency: low. There is no learning-related mapping between gesture and content, the users’ movements are elicited primarily for navigation (e.g. tap for next screen). (3) Sense of immersion: low. The display covers far less than 50% of FOV and borders/real world are always present.

The four conditions in the experiment are summarized and mapped to the degree of embodiment in Table 2. All participants read and heard seven sections of a script on the electric field. Participants in symbols and text (S&T) answered traditional test questions between each section. Participants in Lo-EMB watched seven simulations. Participants in Hi-EMB are able to control the seven simulations with gesture. Participants in Hi-EMB/Narrative controlled the seven simulations with gesture and view short cut scene animations before the simulations. After completing all seven sections, all participants took both traditional and *Wacom* assessments.

**Table 2:** Condition name and degree of embodiment in taxonomy

Condition	Name	Passive or active?	Degree taxonomy	Notes on constructs
(1) Symbols and Text – control	S&T	Passive	1	Deemed a very low embodied condition on all counts; cannot account for whether participants visualize text
(2) Low Embodied	Lo-EMB	Passive	2	Sensorimotor = low Gestural congruency = low Immersion = high
(3) High Embodied	Hi-EMB	Active	4	Sensorimotor = high Gestural congruency = high Immersion = high
(4) High Embodied-Narr	Hi-EMB/Narr	Active	4	Sensorimotor = high Gestural congruency = high Immersion = high

## Being active

One of our main questions is what happens when the lesson changes from low embodied passive viewing (condition 2) to high embodied active (condition 3). One way to move learners out of a passive viewing experience and into a more effortful cognitive state is to make the learner physically move objects and build models on the screen via gestures. If the learner is induced to manipulate the content on screen and control the content with representational gestures that are congruent to what is being learned, we would consider that experience to be high embodied. Because participants are activating associated sensorimotor areas, they may learn the content faster or in a deeper manner. Gestures may provide an additional code for memory; this motor code may strengthen the memory trace, or representation, and add additional retrieval cues. As Goldin-Meadow (2006) posits, gesturing may “lighten the burden on the verbal store” in a speaker’s or learner’s mind. We believe this may make it easier for the learner to perform other verbal tasks, like encoding science concepts. What is being gestured matters as well. Research on a sequence of videotaped gestures showed that watching a tutor give an explanation of a dynamic system accompanied by gestures representing the sequence of actions led to deeper understanding of the system’s operation compared to seeing gestures representing the structure of the parts (Kang & Tversky, 2016).

We include a brief description of the “Scuff-o-meter” simulation, both low and high embodied to highlight the difference. In the low embodied (condition 2) version, the participant watched a prerecorded animation of a charge building up on a virtual hand. In the high embodied active version, participants physically shuffled their feet back and forth on the carpeted floor and the Microsoft *Kinect* sensor registered the movements. The shuffling movement is congruent to, that is, well-mapped to, what occurs in real life as electrons are stripped from the top of a carpet. Rate of accrual of electrons is mapped to the participants’ actual movements and changes the charge on the virtual hand. This is a strong example of gestural congruency (Segal, 2011). In addition, when the participants moved their physical right hand in three-dimensional (3D) space, the virtual hand on screen moved towards the metal sphere for a shock. In the high embodied conditions, there is an added level of contextualization because the gestures and body movements control the screen action and give the participant more agency.

If Goldin-Meadow’s postulation is correct that gesturing helps to offload cognition (Goldin-Meadow, 2011) and free up resources for further processing, then perhaps educational designers should consider methods of teaching science content that make more use of gestures. Gestures require motor planning. It is hypothesized that making a gesture first requires a “mental simulation” before the action and that early stage motor and premotor areas of the brain are activated in action-appropriate ways (Hostetter & Alibali, 2008). This pre-action time is also called the covert state. The covert state of imagining an action appears to stimulate the same collaries or motor areas as overt action, i.e. motor cortex, the cerebellum, and basal ganglia (Jeannerod, 2001). We propose that the greater amount of motor and pre-motor activity associated with gesture during the act of encoding will aid learners at the time of retrieval because the learning signal will have been strengthened. More support comes from recent work with younger learners showing neural differences when children are active versus passive during a learning experience. When 5- to 6-year-old children actively manipulated an object while hearing a new label and then heard the label again, motor areas of their brains were more likely to be activated upon subsequent viewing compared with when they were only allowed to passively watch an experimenter manipulate the named object (James & Swain, 2011). A similar increased recruitment of sensorimotor brain areas occurred when children wrote letters versus when they watch an experimenter write (Kersey & James, 2013). See the Kontra and Beilock work for evidence in the physics domain (Kontra et al., 2015).

STEM (Science, Technology, Engineering, and Math) topics may benefit from being taught in an embodied manner using new media. However, the gestures need to be congruent to the task learned. Koch, Glawe, and Holt (2011) report that participants react faster in a Stroop condition using congruent gestures (up movement attached to word “happy”) compared to incongruent gestures (down movement for “happy”) performed on a large 28-inch slider (Koch et al., 2011). Glenberg and Kaschak (2002) vary the direction of button pushes for sentence comprehension. Congruent sentences were judged faster than the action mismatch sentences. A wide range of topics are now being instructed using theories of embodiment or based on gestures. Abrahamson (2009) researches mathematics and proportionalities. Alibali and Nathan explore learning and teaching diverse math topics including equation solving, word-problem solving, and algebraic and geometric concepts (Alibali & Nathan, 2012; Nathan et al., 2014). Using congruent whole body movements and immersive MR platforms, others have shown increased learning about astronomy (Lindgren, Tscholl, Wang, & Johnson, 2016) and electric circuits (Yoon, Elinich, Wang, Steinmeier, & Tucker, 2012). Virtual worlds are being used to understand spatial maps (Weisberg & Newcombe, 2015) and further body metaphor work is being done with motion capture sensors, like the *Kinect*, to teach students computer coding via dance moves (Parmar et al., 2016). This small sample of embodied research highlights the variety of platforms and methodologies used to explore the positive effects of embodiment on education. Our lab focuses on understanding the best pedagogies available that map to current technologies in today’s classrooms. To that end, many science simulations are being created that are also gamified.

## Game narrative

The distinction between a “simulation” and a “game” is elusive. Is an interactive simulation with a storyline a game? Because well-designed games keep players coming back for more, we wanted to know if “stitching” together short science simulations with a narrative storyline would positively affect engagement and learning. There is a general belief among educators that stories and games keep children interested and engaged (Gee, 2007) in the content to be learned, but this has not been very rigorously tested (or at least published on). In the late 1980s, a framework based on games and intrinsic motivation with four motivating factors was created (Malone & Lepper, 1987), but the simple game in that study has little in common with the games students are now exposed to. With so many game factors and mechanics to choose from, a critical question becomes how to integrate the ones that will result in optimal learning? Recent research shows that adding leaderboards and badges to a semester-long course actually had negative effects on motivation, satisfaction, and empowerment over time (Hanus & Fox, 2015).

We adhere to a classic definition of a game, that it is “a system in which players engage in an artificial conflict defined by rules, that results in a quantifiable outcome” (Salen & Zimmerman, 2004). In that sense, our simulations qualify as games. In all games, it is traditional to have a conflict. However, adding a storified conflict via narrative to a science lesson may not positively affect learning, especially if story comprehension competes with cognitive processing time and resources. We may also discover that not all content is well-suited to a game type format. The approximately 1-h long session in our study consisted of a set of seven short science simulations and it was hypothesized that a story line with a Lightning Master and his mischievous dragon would engage students further and motivate them to maintain effortful attention and processing through the multiple simulations.

We have not found many randomized controlled trials (RCTs) that test the narrative effect on learning with an empirical design. One study on a Flash-based puzzle game for physics found that students preferred to play the narrative version and played more often. However, the post-intervention physics knowledge scores were not significantly different between groups (Marsh et al., 2011). That is, players reported liking the narrative game more, but preference did not affect learning. Two experiments based on work by Koenig et al. (Adams, Mayer, MacNamara, Koenig, & Wainess, 2011; Koenig, 2008) also address the narrative hypothesis. The 2011 study did not reveal greater learning gains for the participants in the narrative condition, i.e. the group given the text-based background and stated goal for learning about wet cell batteries did not outperform the control group. Koenig (2008) found a significant increase in enjoyment of the game in the narrative condition, but only a statistical trend for higher post-test content scores.

The content in our study was taught via a series of instructional text sections that participants were asked to read while an audio recording of the text played as well. The narrative wrapper was delivered after the text and before the simulations via seven comic book style cut scenes that cohered or motivated the seven text sections. In order to understand if adding a cohering narrative made the lesson more engaging and/or affected learning, the storyline was written from scratch. The science education community is very committed to finding methods for adding motivation and engagement to science; it can sometimes be difficult for students to maintain persistence while studying. Persistence is critical for academic success (Duckworth, Peterson, Matthews, & Kelly, 2007). Perhaps adding a narrative storyline will induce a localized persistence, or grit, in some learners. It may ignite a curiosity regarding the end of the story and give learners a reason to carry on with studying and sense-making.

Our comic book-like cut scene graphics set up a dramatic arc storyline with conflict and eventual resolution. The narrative inserts gave rationale for the upcoming simulations, i.e. make balloons stick to walls via induction for an upcoming party. It is known that stories can make content easier to be remembered (Graesser, Singer, & Trabasso, 1994) and we also predicted that a story structure might increase engagement and aid in sense-making. A good story should contain the four Cs (Willingham, 2004): causality, conflict, complications, and character. We added the four Cs in seven extra minutes. However, it might also be the case that a story is distracting. Learners are working with limited cognitive capacity while engaged in new media (Mayer, 2009). A compelling story line may distract, contain irrelevancies, or proliferate in what is termed seductive details (Garner, Gillingham, & White, 1989; Mayer, Griffith, Jurkowitz, & Rothman, 2008). Creating a compelling narrative is effortful for the content creators and can involve substantial extra resources (e.g. artwork, programming, storyline scripting, etc.). The narrative effect is a timely research question and a definitive answer regarding its value could save considerable time and funds down the line for other designers and researchers.

## Test sensitivity

Whereas adding narrative and more embodiment may lead to greater learning, that does not necessarily imply that that learning can be demonstrated on a standard, pencil and paper/keyboard verbal type of test. E.g., a student may have learned that similarly charged particles negatively accelerate as they move apart, but that knowledge may be motoric and spatial and not easily conveyed by words. Instead, an assessment procedure that taps into that motoric and spatial knowledge may provide a more sensitive measure of knowledge gained. Thus, we designed a more embodied test using a very large tracking pad, the Intuous *Wacom* Pro. With the large *Wacom* pad, participants could place a finger directly on a surface that afforded a 15.1-inch drawing diagonal. The *Kinect* sensor was not used as a post-intervention assessment device because two out of the four experimental conditions had experience with the sensor and they would have had an advantage. The *Wacom* also served as an uncompromised embodied transfer measure.

To comprehend abstract content like the electric field, one must truly understand how charges move and the meaning of vectors. In two of the experimental conditions participants were able to use larger arm and hand movements (i.e., swiping the hand from chest level up to a full extension above the head would be one of the biggest gestures) to create vectors in 3D space. We predicted that the two groups that used gross body movements during learning to create vectors and other representations would do better on the assessment methodology that facilitated larger gestures and movements. However, it may also be the case that watching large vectors being animated on a screen may be just as effective in priming users’ sensorimotor areas associated with the gesture. If that is true, then condition 2 (the low embodied, view-only condition) would show the same gains on the *Wacom*-driven post-test as the two high embodied conditions. The *Wacom’s* roomy drawing area encouraged participants to draw vectors and also “be the charge.” They could move their finger over a large surface and demonstrate how a free charge might move through the electric field. This type of assessment is more haptic in a hands-on manner and more “immediate” because the human finger touches a surface rather than the fingers grasping a mouse and the mouse movement affecting the interface display. The *Wacom* measure may hold more embodied ecological validity than mouse-driven measures.

## The topic and predictions

More on the electric field as the topic can be found in Appendix 2. Briefly, we wanted an abstract topic that would include motion and effects that are not seen with the naked eye, because mixed and virtual realities are well-positioned to make the unseen seen. The electric field would be new, or at least partially forgotten, for many of the psychology students in this experiment. Because it is a difficult topic, there were few concerns about ceiling effects. Coulomb’s Law^1^ and understanding forces that act at a distance are in the Next Generation Science Standards and are recommended to be taught in all U.S. high schools.

The four research question and the predictions are:

- R1: *Simple embodiment*. Is learning affected by whether the content is primarily symbolic or embodied? We predict better learning in the three embodied conditions compared to S&T.
- R2: *Gestural boost*. Is learning further affected by whether active gestures are added to the learning session? We predict better learning in Hi-EMB and Hi-EMB/Narr compared to Lo-EMB with passive viewing.
- R3: *Game narrative*. Are learning and amount of engagement affected by whether learners are presented with a narrative storyline that relates and coheres the multiple simulations? We predict better learning in Hi-EMB/Narr compared to Hi-EMB.
- R4: *Test sensitivity.* Are differential learning effects revealed by different types of assessment procedures? The prediction is that different learning will be revealed by assessments that are more closely aligned with the methods of encoding. We predict that the *Wacom* tablet test, compared to the keyboard test, will reveal greater learning gains for the high embodied conditions.

## Methods

### Participants

A total of 166 undergraduate students (74 women, 92 men) from a large University in the United States participated in a Psychology 101 study for 2.5 h of course credit. Inclusion criteria included being able to stand for 1.5 h. Participants were randomly assigned to the four conditions after signing informed consents. If they arrived with extremely low expressive English skills, the tester could choose to administer an experimenter-designed language test wherein the participants read a paragraph in English and verbally answered five questions. One hundred and seventy-two students came, but six were dismissed from the study, with credit. Because there was a non-trivial amount of reading in the assignment, it was crucial that participants be able to read and comprehend the English language load. The demographics survey revealed that 27 participants (16%) took the TOEFL test and that 30% were science majors.

### Apparati

Two intervention rooms were used. Both had equal-sized large projection surfaces. The first room had a Promethean™ *ACTIVBoard* with a 78-inch diagonal, the second room had a ceiling-mounted NEC™ M300WS projector that projected onto a white wall with a 78-inch diagonal display. Both projection devices and CPUs connected to the Microsoft *Kinect* (Version 1 or “Xbox 360”) sensor. In the first two conditions the *Kinect* sensor was disabled (S&T and Lo-EMB) but visually present.

The Intuous® *Wacom Pro* multitouch tablet was used to gather the gestures as one of the tests. The one tablet was shared between the two test rooms due to cost. The *Wacom* is the go-to drawing surface for artists due to its pressure sensitivity. The *Pro* has a physical size of 19.1 × 12.5 inches; however, the *Pro* active area (sensitive to finger touch) was 12.8 × 8.0 (i.e., a 15.1-inch diagonal).

### Design

The study was a mixed 2 × 4 design. The first factor was time with a pre-test and post-test and the second factor was embodiment/narrative with four conditions. Participants were randomly assigned to one of four conditions via a random number generator. An experimenter worked with a single participant one-on-one. A full session with all the tests took an average of 75 min (the time on task, or “instructed content” lasted on average of 50 min in the first three conditions and 57 min in the final narrative condition). We note that four of the non-native speakers took over 2 h (120 min) to complete all the tests and content.

Every section began with instructional text cards on aspects of the electric field. Participants were asked to read the short cards; however, the cards were also delivered auditorally. Participants could not skip forward with the clicker through the instructional cards until the cards had been heard through. The instructional text was written to be very low embodied, for example, words with agency and emotion were avoided, so the words “push, pull, attract, repel” did not show up, instead, terms like “moves towards” or “moves away from” were used. The instructional text did not vary between conditions. All participants stood in the middle of the room and advanced to new sections with a handheld clicker. In this way, pacing was somewhat under user control. Although they could go back and reread within a text section, they could not skip to entirely new (or old) sections. There were seven sections in the lesson. The manipulation is what happened in between the instructional text cards.

The Manipulated Conditions.

**Fig. 1:**
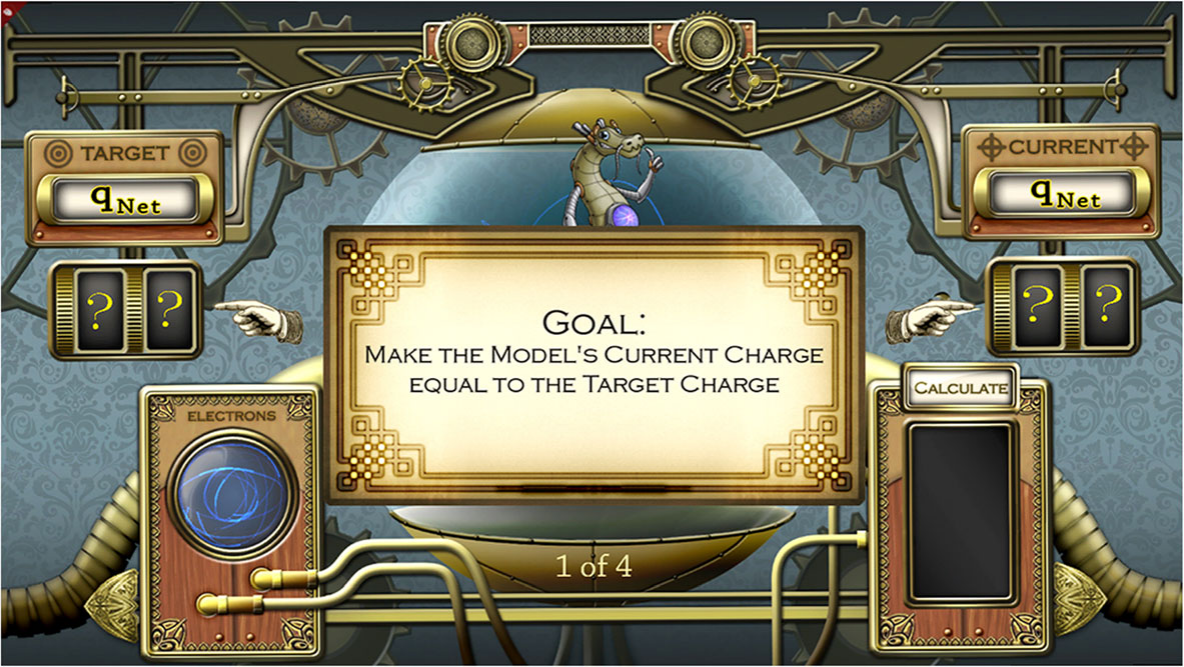
The Electron Counter screenshot with stated goal

**Fig. 2:**
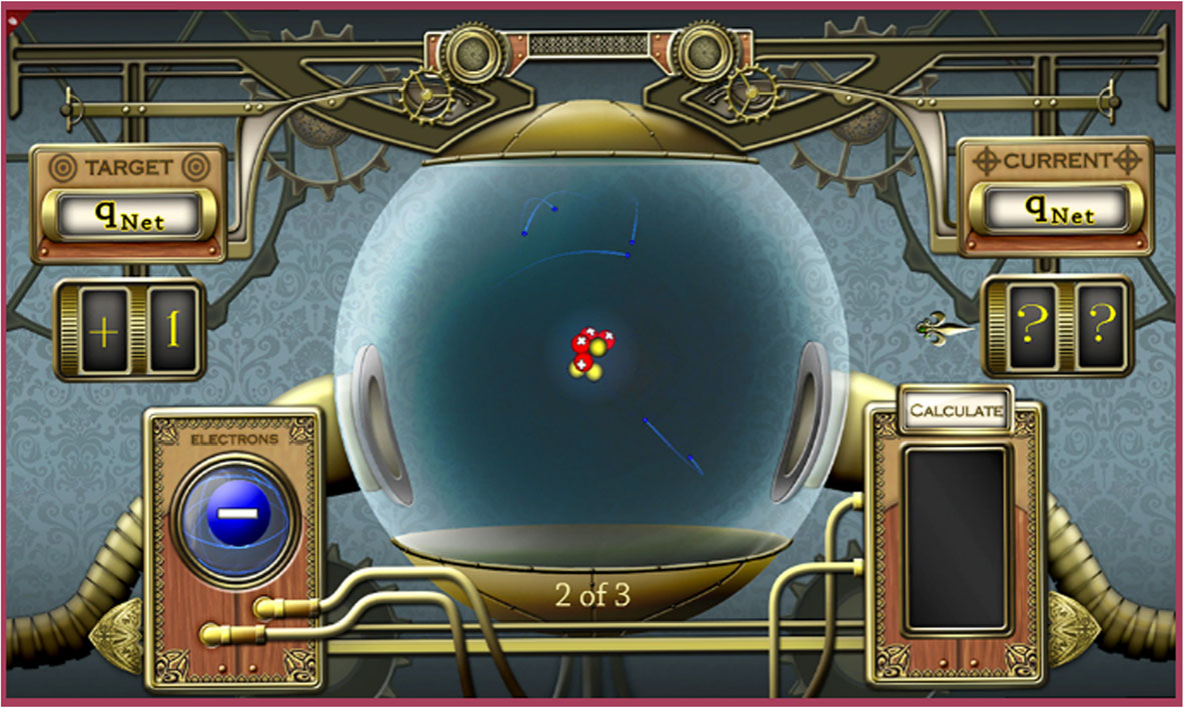
The Electron Counter simulation with central counting sphere

**Fig. 3:**
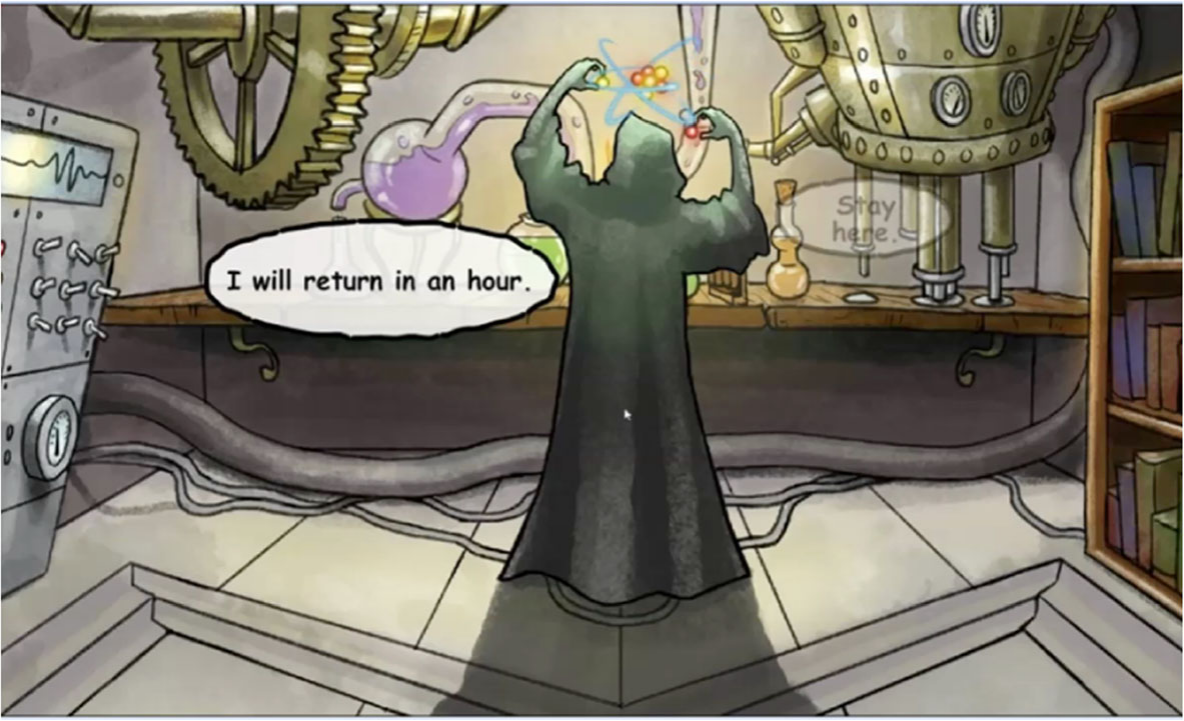
Inside the Lightning Master’s lab, sample cut scene

1. *Symbols and Text (S&T)*. In between the text card sections, the S&T group answered quiz questions that included only text and symbols for equations and questions. Participants read the short multiple choice text-only questions that appeared after each content section. After each text section there were four multiple choice questions designed to reinforce what had just been read and to equate for time between conditions. Thus, no graphics nor simulations were seen or acted upon between sections, participants only answered quiz questions and received feedback after the submission of each answer. We equate this condition to the sort of textbook style of learning prevalent until about a decade ago. In all conditions participants received real-time feedback on submissions.
2. *Low Emb*. In the low embodied condition, participants watched animations of simulations that were pre-created (like viewing a video). The participants could start the animations, but they could not actively control the action within the animations. As an example, in the Electron Counter, they watched seven trials of electrons being added or deleted from the counting sphere (behind the GOAL card in Fig. 1). They then saw the sum calculated in real time via moving arrows on the bottom right in the Calculate box. See Fig. 1.
  In the low embodied condition, they did not perform the action of moving their hands to “grab” the electrons; they observed an animated hand on screen doing that action. The first three trials were “show trials.” We scaffolded how the simulation worked; the show trials always included at least one error that received feedback. The next four trials were for a score and were view only. Again, the first three conditions (1, 2, and 3) were equated for time.
3. *High EMB*. The final two conditions (3 and 4) are both considered high embodied. In condition 3, the *Kinect* sensor was turned on. The *Kinect* sensor was present in the experimental rooms in all four conditions, but only activated for conditions 3 and 4. After the instructional text sections, participants were able to physically interact with the seven simulations (described below). As an example, in the Electron Counter, the *Kinect* read the location of the “highest hand” at 60 Hertz. Using this hand algorithm, it was not necessary to worry about handedness. Participants were told to raise their dominant hand with a clicker and press the button to select electrons from the holding area in the Electron box (see Fig. 2). After viewing three practice trials (similar to condition 2), the participants then took control of the next four trials in which they actively grabbed electrons and created their own atoms. Participants could grab electrons from the bottom left and add electrons into the sphere. Or, if the atom had too many electrons in the nucleus, participants could click and remove electrons from the atom. When participants decided they had added or deleted the correct amount of negative electrons to match the target value, they then selected *Calculate* with the clicker. A tally was then displayed in *Current* with a moving arrow that summed up the negative (electrons) and positive (protons) charges to reveal q _net_. If *Current* matched the *Target* value then CORRECT feedback showed up (see video at www.embodied-games.com to clarify the sequence or view the Youtube at https://www.youtube.com/watch?v=eap7vQbMbWQ).
4. *High EMB-Narr (with narrative story line).* Condition 4 was the same as the condition 3 except that seven graphic narrative cut scenes (see Fig. 3) were inserted before the simulations. This figure shows the Lightning Master’s lab. A cut scene is a comic-style graphic with text bubbles that appeared and faded; ours were accompanied by music. The total time of display was 418 s (referred to as 7 min hereafter). The cut scenes were displayed after the instructional text and motivated the next simulation. The participant’s point of view (POV) was a first person in the role of an “apprentice to the Lightning Master.” The seven cut scenes are further described in the procedure section.

### Procedure

Participants affirmed they could stand for up to 1.5 h, though usually the standing portion only lasted for 50 min. The order of tasks was the same for all four conditions:

- Participants signed consent forms and were randomly assigned to condition. Based on the few minutes of conversation with the experimenter, participants may have taken the 3-min long English reading test.
- Content knowledge pre-test – traditional keyboard. This was a non-gesture-based assessment using the keyboard as the input device. See Additional file 1.
- Content knowledge pre-test – gesture-based. This was a gesture-based assessment that used the *Wacom Intuous* Pro tablet. See Appendix 1.
- Intervention – All participants stood in the center of the test room, 5 feet in front of the large display. With a clicker, they were able to advance to sections at their own pace. They were seated after the intervention.
- Engagement survey – On the computer, participants answered several engagement questions.
- Content knowledge post-test – Traditional keyboard. Participants took the same pre-test keyboard-based questions.
- Content knowledge post-test – gesture-based. Participants took the same pre-test *Wacom-*based questions.

#### The instructional text

The text on the instructional text cards did not vary between conditions. Participants would silently read and listen to instructional text cards and they could skip backwards to reread within a section. The text was written to be very low embodied, that is, no references were made to the body and no anthropomorphisized expressions were present. After each text section, participants were asked to type in what they learned with open text. Those analyses will be reported elsewhere. The main content concerned charge carried at a distance and the electric field. This is related to Coulomb’s law^2^; for this study we focused on the proportionality.

### The seven simulations

**Fig. 4:**
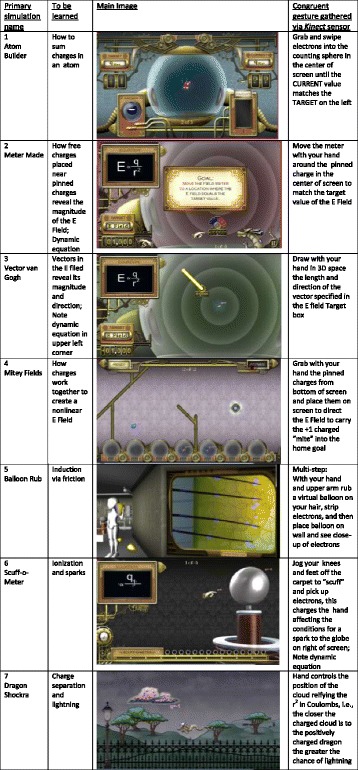
Image Table with main screen shot describing the science simulations

Each of the seven simulations was created in two versions (total = 14): a passive view-only version for condition 2 (Low Embodied) and the manipulable generative version for the two active conditions: conditions 3 (High Embodied) and 4 (High Embodied-Narr). Appendix 2 contains a detailed description of the 14 minigames and their feedback. Below is a shorter description of the low embodied version (A) followed by the high embodied description (B). Figure 4 is a table with seven key images that represent the main screen image for the simulations.

Simulation 1: Atom Builder

To be learned: How to sum charges in an atom

(A) *Atom Builder Low Embodied*: This simulation served to remind players of the structure of an atom and how charge is measured. In the center of the screen was a slowly spinning nucleus (with protons in red and neutrons in yellow). The goal was to match the target number for valence and an animation either added or deleted electrons to reach the target q_net_ (displayed in upper left corner). For both the low and high versions of Atom Counter, there were seven trials total.

(B) *Atom Counter High Embodied*: The *Kinect* sensor was always in front of the screen in all the conditions. The adding and deleting of electrons was controlled with the player’s highest hand. If the participant held his/her hand over the *Electrons* box (bottom left of Fig. 2) or the central counting sphere and hit the advance button on the clicker, then the electrons would stop spinning and one electron would glow. The participant was then able to “grab” and move the glowing electron around on the screen. The electron would be released when the participant released the clicker button. This simulation is also described in the introduction section.

Simulation 2: Meter Made

To be learned: How free charges placed near pinned charges reveal the magnitude of the E field, includes dynamic equation

(A) *Meter Made – Low Embodied*. This simulation was designed to help learners understand that the strength of the electric field (E field) can be assessed with a meter at one point in space. The meter has a charge of +1. The goal is to place the meter, currently filled with question marks in the second image in Fig. 4, so that it will match the *Target* E field (bottom left of screen), currently 1.000. In the middle of the screen is a pinned charge. The pinned charge will vary in valence and magnitude with each trial. The end game goal is to match the *Target* E field which reads 1.000. The participant watches the blue and red meter as it moves around the screen to the correct location where the E field is equal to 1.000.

(B) *Meter Made – High Embodied*. The placement of the meter is controlled by the participant’s highest hand, s/he then presses the clicker button when ready to place the virtual meter on screen. The E field measurement number changes dynamically as the meter is moved. Error feedback was similar to that in the low embodied condition, three trials are allowed before a hint appears. See image number two in Fig. 4.

Simulation 3: Vector Van Gogh

To be learned: Vectors in the E field reveal its magnitude and direction, included dynamic proportionality

(A) *Vector Van Gogh – Low Embodied*. This simulation was designed to help participants understand the concept of vectors as possessing both magnitude (length of the arrow) and direction (the direction connotes attraction or repulsion). Participants are able to further explore how the strength of the E field can be assessed with pinned and free charges. The participant would watch vectors being drawn from a circular “start point,” a dynamic measurement was displayed under the start point. See image number three in Fig. 4.

(B) *Vector van Gogh – High Embodied*. The *Kinect* was used to track the highest hand. The clicker was held in the highest hand. The goal of the high embodied version was for the participant to draw in the air the correct length and direction of the vector. When a participant would start to draw a vector the forward button was held down on the clicker and that button was released when the vector was finished. Similar to the low embodied version, this version also contained two levels of scaffolding; vectors were first animated as show trials, then they were generated (either as a video or by self) and scored.

Simulations 4a and 4b: Push Me Pull U and Mitey Electric Field Hockey

(A) and (B) *Push Me Pull U*. This served as an observational warm-up to explore vectors associated with two atoms. The dynamic equation in the upper left corner now includes a numerator where q_1_ is multiplied by q_2_. The participants would click *Activate* at the top of the screen and observe how two charged particles would react in a contained space. Both particles would be released from a pinned situation at once and depending on their magnitude and valence, they would either head towards or away from each other. There were four examples.

Simulation 4: Mitey Fields

To be learned: How charges work together to create a non-linear E field

(A) *Mitey Fields – Low Embodied Version*. Participants observed four simulations in this version. An animation showed how pinned charges could be placed on the screen from the holding spheres below, see the fourth image in Fig. 4. The pinned charge, for example, the q = –5 charge, would be animated up from the lower screen area and placed in the middle of the screen. Once *Activate* was hit, the resultant E field would carry the blue creature called the “mite” back into a hole. Two errors were modeled as well. The mite always had a charge of +1.

(B) *Mitey Fields – High Embodied*. In the high embodied version, the participants were able to use their highest hand and the clicker to grab charges from the holding spheres on the bottom of the screen and pin the charges anywhere on the screen. The mite is always charged with +1 so placing the –5 charge behind the mite will make it head straight into the hole. After three errors on-screen hints were given. Videos of all simulation can be seen at the main website, but this game is now a stand-alone one and can be downloaded https://www.embodied-games.com/games/all/mitey-fields.

Simulation 5: Balloon Rub – Friction and Induction

To be learned: Induction via friction

(A) *Balloon Rub – Low Embodied*. This simulation addressed two important topics. The first topic was friction and it was demonstrated with the classic rubbing of a balloon on one’s hair. To try to mitigate race and gender issues, a stylized artist’s mannequin (avatar or manikin) was used to represent the body on screen. On screen, a yellow balloon was rubbed up and down the side of the avatar’s head to demonstrate how electrons can be stripped from hair (see the fifth image in Fig. 4). As the yellow balloon picked up more electrons the balloon side touching the hair turned to blue, this simulated the balloon becoming charged with electrons from the hair.

**Fig. 5:**
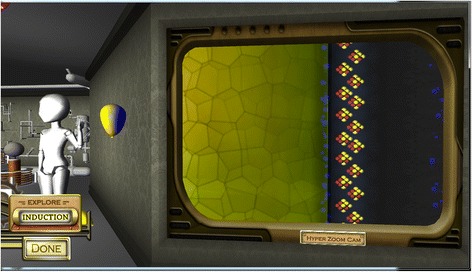
The negative electrons on the balloon push the negative electrons on the wall deeper into the wall, so the balloon can bond momentarily with the slightly more positive wall surface

The right portion of the screenshot is labeled “Hyper Zoom Camera.” The black strands represent individual hairs and the blue particles are electrons with a charge of –1 each. The second topic of induction was introduced as an animation wherein the avatar pushed the balloon towards the wall and the balloon then stuck to the wall. In Fig. 5, the participant was able to see, subatomically, how the electrons on the balloon surface interacted with the electrons in the wall. In the Hyper Zoom shot, the yellow balloon side is speckled with extra blue electrons, and on the right side (in the wall) the blue electrons are balanced in the neutral wall.

Figure 5 shows the state a few seconds later when the balloon is stuck to the wall. Via induction, the extra electrons on the balloon’s surface have pushed the electrons closest to the surface of the wall a bit further into the wall. The balloon’s negative surface is now strongly attracted to the positive protons near the wall’s surface.

(B) *Balloon Rub – High Embodied*. In the high embodied version, the *Kinect* sensor tracked the participant’s right arm movements. Participants faced the screen and sensor, and were instructed to pretend they were rubbing a balloon on their hair. As the participant’s right wrist joint moved up and down, the algorithm gathered the ratio of that movement to map to the velocity of the avatar moving the virtual balloon up and down on screen, i.e. the avatar on screen mimicked the participant’s right arm movements. The velocity of the participant’s balloon rub movement was used to apply force to a physics simulation of hair strands in the Hyper Zoom shot, the hair strands also moved in rhythm to the participants rubbing motion. There was therefore a large degree of agency associated with this simulation. For the second topic of induction, when the participant straightened out his/her right arm, the mannequin’s arm would also straighten out and move the virtual balloon towards the wall. The participant could then leave or retrieve the balloon from the wall.

Simulation 6: Scuff-o-meter

To be learned: How friction can strip electrons from a surface and the potential difference between two charged objects can induce a spark

(A) *Low Embodied – Scuff-o-meter*. In the low embodied version, the participants watched four animations of a spark occurring between the virtual hand on screen and the silver “glow globe” or spark candle on the right. The glow globe appeared with a different charge in each of the four trials. In the sixth image in Fig. 4, the glow charge is set to *q* = 10. In the low embodied animation version, the hand on the left side of the screen would animate back and forth rapidly showing that it was picking up electrons via friction (similar to the balloon simulation). The charge on the hand increased with each scuff back and forth. The dynamic formula on screen helped learners to deduce the relationship between the build-up of electrons (the q) and the distance needed for a spark (the r).

(B) *Scuff-o-Meter – High Embodied Version*. In the high embodied version, the *Kinect* was used to track the user’s highest hand, as well as the positions of the two knee joints. First, participants were instructed to scuff, that is shuffle, their feet back and forth along a 2-m-long strip of the carpeted room. Participants could see on screen how many electrons they accrued as they scuffed back and forth. They could see electrons accrue both on the virtual hand (via the “q=” label) and as the blue dot electrons increasing in the circles on bottom of the screen (the Scuff-o-meter). When participants decided they had gathered enough electrons to create a spark, they brought their human hand, which was mapped to the virtual hand, towards the virtual glow globe for a spark.

Simulation 7: Dragon Shockra!

To be learned: Charge separation and some of the conditions for lightning

(A) *Dragon Shockra – Low Embodied*. In the low embodied version, the participants were told that they would see a simulation where pieces of equipment would be “zapped” from a flying dragon, points would be awarded when pieces were knocked off. Participants should “notice the correct conditions” that preceded a lightning strike. To wit, the q_net_ in the cloud would need to be high enough and the dragon would need to be close enough for a lightning strike. The negative electrons would dynamically accrue in the bottom of the cloud and the charge at the bottom of the cloud was tallied as q_net_. See the seventh image in Fig. 4.

This was a “scrolling runner game.” The foreground would scroll to the right and the dragon would appear to fly to the left, towards the cloud. The dragon simulated quasi-random movements. (See Appendix 2 for a further description of all game algorithms.) The dragon had a charge of +1. The r, or distance, of the dragon to the cloud was an important variable that effected when the lightning strike would occur, players were encouraged to watch the interaction between charge and distance. Participants observed the 3-min animation that resulted in the dragon being struck three times. In the view-only condition, trees were also struck; that is, mistakes were also modeled.

(B) *Dragon Shockra – High Embodied*. As in the previous condition, the cloud location was constrained to move within the top left quadrant of the screen (counted as 100 units vertical from top left corner). The seventh image in Fig. 4 shows the cloud in the far bottom right position. In the high embodied version, the *Kinect* was used to track the participant’s highest hand. The participant’s hand position controlled cloud location. Once the timer started the three minute countdown, the dragon would automatically “fly” toward the left edge of the screen (begin scrolling). The foregrounded fence and light poles scrolled to the right giving the illusion of the dragon flying. The participants controlled how close the cloud could get to the dragon. The dragon’s flight path was perceived as “quasi-random.” The players deduced they should not simply position the cloud to always be low in the sky, because if the cloud were highly charged and low, it would strike the closest positively charged object. That object would sometimes be a positive tree. Similar to version A, when trees were hit with lightning this was deemed a mistake; the trees would smolder and the cloud reset to a neutral charge wasting game time. The play mechanic was designed so that participants could use their knowledge of Coulomb’s law to be strategic and win more effectively. It was important to not waste too many strikes within the three minute time limit. If players knocked all three pieces of equipment off the dragon before the time limit, the game still continued for the full 3 min to equate for time on task between conditions.

### The narrative story line

Now that the simulations have been described, it will be more meaningful to describe the cut scene narratives that preceded each simulation in condition 4, Embodied with Narrative.

i. *Before Electron Counter*. The Lightning Master is leaving for 1 h but encourages you (the player referred to as the apprentice, but always off screen) to keep working to understand the electric field. After the Master leaves, a mischievous dragon in a cage informs you it is the Master’s birthday and asks to be let out to start decorating for the party. Will you let the dragon out of the cage?
ii. *Before Vector van Gogh*. The dragon encourages the apprentice to learn as much as possible about vectors because it will help them prepare for the party. For example, to light the glow spheres—that are like candles—you will have to know about sparks and the E field.
iii. *Before Meter Made*. The dragon encourages the apprentice to understand charges as the knowledge will help get the “vector machine ready.”
iv. *Before Push Me-Pull U* and *Mitey Fields*. The dragon is happily flying around the lab and knocks over a glass sphere holding the “mites.” These mites have a charge of +1. The mites need to be captured and returned to another sphere. It is your job to use the E field to guide the blue mites.
v. *Before Balloon Induction*. The dragon is seen hugging the vector machine with hearts flying out. He reiterates the importance of understanding induction, and encourages you to get back to your studies.
vi. *Before Scuff n Spark*. The dragon tells you to find a way to put balloons up on the wall as decoration for the upcoming party.
vii. *Before Dragon Shockra*. The Lightning Master has returned and sees an open window. The Master realizes the dragon has escaped and says, “Don’t worry, this has happened before.” The next scene shows the dragon is out in the sky wreaking havoc as he supercharges all the barns and houses outside in the field. The supercharged dragon must be captured.

### Measures

Content knowledge and level of engagement were assessed. Content knowledge was assessed with two different measures given as invariant pre-tests and post-tests. Engagement was measured only at post-intervention.

#### Content knowledge test: computer version

The Electric Fields Test was created by a team of three physics instructors and was piloted on five age-appropriate participants. The study version is included in Additional file 1. It was administered on *Survey Gizmo*, only one question appeared at a time. The same version was given at pre-test and post-test with no feedback.

There were 34 items on the test. It started with a simple refresher “fill in the parts of an atom” and ended with complex questions about charge movement. Items were: 14 multiple choice questions, six Cloze tasks that required one or two word responses, and 14 short answer prompts. A rubric was created to score the short answers and scores of 0 to 3 were awarded. As an example for question 21: “Imagine a cloud hovering above the desert. The bottom of the cloud is negatively charged. The surface of the earth is positively charged. Suppose we place a positively charged particle and a negatively charged particle in the air between the cloud and the surface of the earth. What will happen to the negative charge?”

- 3 points = It will move with increasing speed (any word to connote “acceleration”) away from the cloud and towards the earth
- 2 points = move toward the earth and away from the cloud – *correct direction only gets 2 points.*
- 1 point = Move in one direction – unspecified
- 0 points = incorrect – move towards cloud, not move, or DK (“don’t know”).

The maximum possible score for the test was 102 points. There were no ceiling issues; the participants’ scores were in the range of 7–74.

#### Content knowledge test: gesture-based Wacom version

One of the research questions concerned whether knowledge gain differences would be seen using an assessment platform based on gestures. The *Kinect* was not used to gather body movement because only half of the conditions would have been familiarized with that system by post-test. Instead, a large format tablet that was novel for all the participants at that time was chosen, the *Wacom™ Intuous Pro* (15.1-inch or 38.4-cm active diagonal). To understand the electric field it is crucial to understand vectors and how charged particles move in the field. Our *Wacom* test focused on how particles move when carried by the E field and contained 11 items. The first three items were simple practice tasks (e.g. draw a vector that is 4 units long).

All participants confirmed they had never used a *Wacom* before. This is essentially a large tracking pad with great sensitivity to and accuracy for touch. For this test phase, the keyboard was moved to the side and the *Wacom* was placed on the table beneath the 16-inch diagonal computer monitor. To keep the assessment as haptic and embodied as possible the stylus was not used, instead participants sat and drew with a fingertip on the *Wacom* surface.

**Fig. 6:**
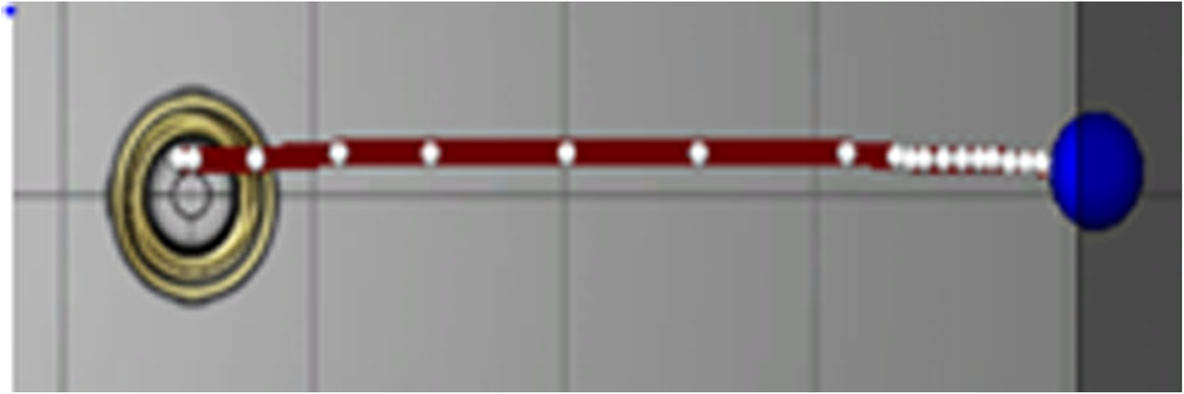
Close-up of acceleration in a *motion map dot trail*. Notice how the *white dots* get closer together towards the end of the finger swipe connoting negative acceleration

In Fig. 6, the placement of the finger was stylized by the large blue circle, as the finger moved a trail was left behind. The participants viewed their motion results on the computer monitor placed at eye level. So, as the finger moved across the *Wacom*, users saw a colored line trailing behind the blue circle. Every 100 ms white dots were placed inside the colored line (see Fig. 6).

This is similar to the motion map concept used in Modeling Instruction (Hestenes, 1987). The placement of the white dots is a visual cue for speed. Users should be able to feel if they are accelerating, but the visual feedback of the white dots as a motion map also allowed users to see that when the finger moves faster the dots spread further apart. In Fig. 6, the dots get closer together as the user is slowing down before stopping on the far right. If participants were not satisfied with the line or vector they had produced, they could tap the “reset” button on the bottom left of the screen, otherwise they would tap “submit.” The system also tallied number of resets. After the practice questions, the eight substantive questions were asked and they were worth seven points each (maximum = 56).

**Fig. 7:**
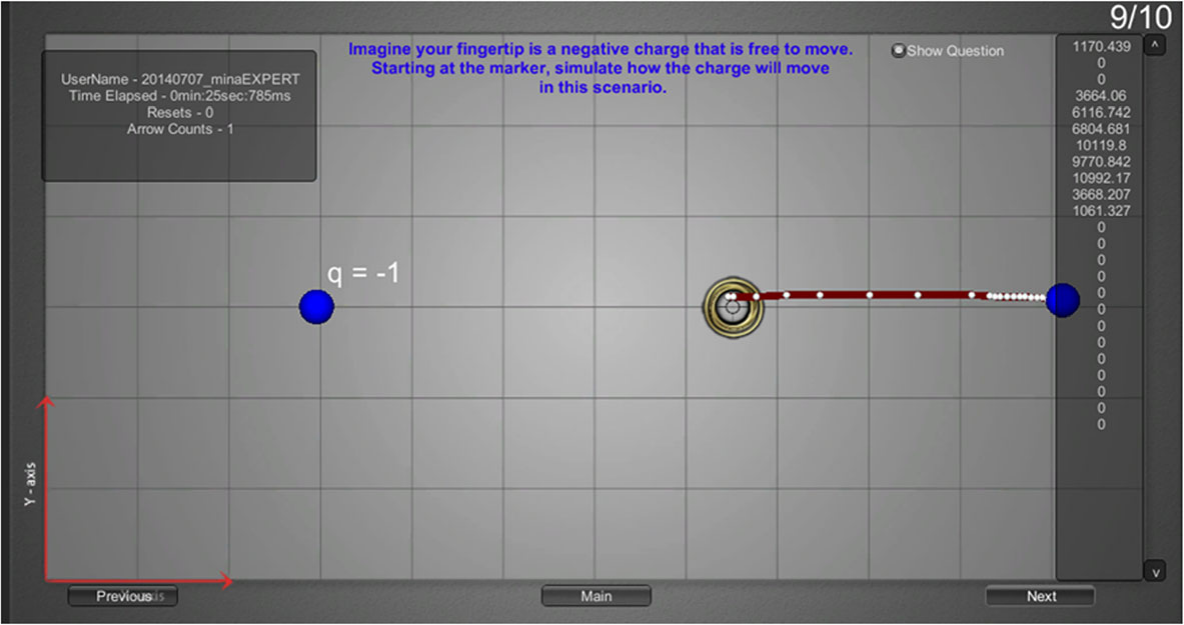
Expert answer to question 6 on the *Wacom* measure

To score, expert vectors were created. Figure 7 shows an expert answer to question 6 that required repulsion (the finger-generated red line should move away from the –1 pinned charge). In addition, negative acceleration should be seen the correct answer as the particle moves further from the pinned charge. In this example, 3 points would be awarded for correct direction and 4 points for showing negative acceleration. We see evidence of negative acceleration in Fig. 7 because the white motion map dots get closer together as the free particle (i.e. the finger tip) moves further from the pinned charge.

The scoring schema was devised by two physics instructors and a computer scientist. They settled on a hybrid type of scoring that was partially automated. A random half of the data was also scored by a graduate student who was trained in the scoring, but blind to condition. The last dot point was always thrown out because pilot participants reported they felt obligated to slow down when reaching the edge of the tablet.

A Guided User Interface (GUI) was created to assist the human scorers and software was designed to score where it was possible to automate. The first two constant velocity questions were the easiest to score, the distance between the dots every 100 ms was gathered and variance in the dot trail was calibrated for equal thirds of the trail. If the variance between the three sections (beginning, middle, and end) varied by more than half of the participant’s individual SD, then the movement was not considered constant. For questions 3 to 6 which dealt with negative and positive acceleration, straightforward answers were harder to achieve. Some participants left multiple dots that could just be eyeballed, but some participants were “rapid drawers” and left only five or six usable dots on the screen. Here, the GUI program helped visualize and quantify the items. It was possible to partition the shortest dot trails into even finer bins, down to 40 ms. A rule was set that a minimum of seven dots were needed (this excluded two participants). The trail was then cut in half. The variance in the first half was compared to the second half. However, this was not always a satisfactory method because some participants would demonstrate acceleration closer to the final quarter of the line and we were unable to define a set algorithm to adequately address these idiosyncrasies. The majority of responses could be scored with the algorithm and agreed upon by the second scorer, but the first scorer set aside a pile of “uncertain” dot trails and removed all information on condition. Then two other scorers needed to come to consensus on those trails. Direction was worth three points and presence of acceleration worth four more. Approximately 8% of the acceleration answers were scored this way. Thus, a consensus between the three scorers was needed before a score was entered into the dataset.

**Fig. 8:**
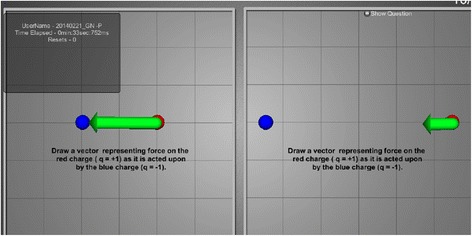
Expert answer to questions 7 and 8 which appeared on same screen. Most important is that vector in the final question (right-hand panel) is shorter than the vector in the left-hand panel

Questions 7 and 8 appeared on the same screen during the test so that a direct comparison could be made. There were no dot trails shown as these were vectors. Again, direction was worth 3 points and now magnitude (vector length) was worth the final 4 points. In question 7, the goal was for the participant to draw a vector showing the force on the red charge (the positive ion on the right-hand side) as it was acted upon by the blue charge. We do not care exactly how long the first vector is in question 7, it just needed to be longer than the vector drawn for question 8. The answer to question 8 was scored in the following manner: 3 points for direction, 3 points for the vector being shorter than the one in question 7, and 1 extra point if the vector was exactly one-quarter the length of the first vector drawn in question 7. Figure 8 shows a participant who did this correctly.

#### Measure-Engagement survey

After the *Wacom* test, the engagement survey was taken on a computer using the *SurveyGizmo* package. The first set of questions were Likert-style ranging from 1 (Strongly disagree) to 5 (Strongly agree).

1. I am now more interested in Electric Fields.
2. The activity was boring. (*Reserve coded.*)
3. I found the activity engaging.
4. I wanted to complete this activity.
5. Overall I found this learning experience to be worth the effort.

The low and high embodied groups were then asked to rank, using 1 through 7, the games they “most enjoyed.” A list of the games was presented and they placed numbers beside the games (simulations).

## Results

### Content knowledge: keyboard assessment

The content knowledge keyboard tests were scored by two trained researchers who were blind to condition. A random sample of 96 items was scored by both testers, revealing a significant correlation (*r* = 0.91, *p* < 0.001). Results are reported only for those participants who completed both pre-test and post-test (n = 166). A one-way analysis of variance (ANOVA) run with SPSS v20 demonstrated that the pre-test scores did not vary by condition, *F* < 1.0.

A linear regression analysis predicting posttest scores was conducted, using Helmert contrasts. The first model of the regression included only contrast 1 (condition 1 [S&T] versus the embodied conditions [2, 3, and 4]). The second model added contrast 2 and asked the low versus high embodied question (i.e. condition 2 [Low Emb] versus conditions 3 and 4 [High Emb and High Emb-Narr]) and contrast 3 which asked the narrative effect question (condition 3 [High Emb] versus condition 4 [High Emb-Narr]). The first model (using only contrast 1) was a significant improvement over the simple model, *F* (1,164) = 4.23, *p* < 0.042, accounting for 2.5% of the variance in post-test scores. This demonstrates that the three embodied conditions performed on average better than the S&T control condition. See Table 3 for the descriptives of the keyboard assessment. The second model (using contrasts 1, 2, and 3) was not a significant improvement over the first model, *F* (3,162) = 1.58, *p* < 0.20. This demonstrates that the low embodied condition did not perform worse than the average of the two high embodied conditions, nor was there a significant high embodied versus high embodied plus narrative difference.

**Table 3:** Means scores for post-test content knowledge with keyboard

Condition, n	Pre-test *M* (*SD*)	Post-test *M* (*SD*)	Grand mean score (contrast 1) *M* (*SD*)	Grand mean effect size, contrast 1 (Cohen’s *d*)
1 S&T (n = 39)	32.4 (11.4)	44.8 (14.1)	44.8 (14.1)	0.38
2 Low Emb (n = 45)	34.2 (12.2)	48.5 (12.1)	49.4 (11.6)	
3 Hi Emb (n = 43)	33.0 (10.5)	49.3 (11.7)		
4 Hi Emb-Narr (n = 39)	36.0 (12.4)	50.5 (11.1)		

### Content knowledge: *Wacom* and Gesture

Results are reported only for participants who completed both pre-test and post-test. There were some technical issues associated with the tablet. In addition, the data on six participants were lost due to data being saved in a wrong file in the beginning of the study (*n* = 134). A one-way analysis of variance (ANOVA) revealed no significant difference among conditions at pre-test, *F* (3, 130) = 1.61, *p* < 0.19.

A linear regression analysis with contrasts was created. Because we were interested in whether being active and using gestures affected performance on the gesture-based *Wacom* test, a model was created with 0, 1 "dummy" contrasts to address S&T + passive versus active embodiment. That contrast was significant, F(1, 132) = 3.77, *p* < 0.05. That is, a contrast that compared condition 1 (S&T) and condition 2 (Low Emb) (the passive viewed content) with the two active high embodied conditions (3 [High Emb] and 4 [High Emb-Narr]) was statistically significant (see Table 4).

**Table 4:** Descriptives for post-test content knowledge with *Wacom*

Condition	Pre-test *M* (*SD*)	Post-test *M* (*SD*)	Grand mean gain score (post – pre) *M* (*SD*)	Effect size (Cohen’s *d*)
1 S&T (n = 27)	28.6 (5.5)	31.1 (8.0)	2.03 (7.03)	0.35
2 Low Emb (n = 32)	30.4 (7.7)	32.1 (8.6)		
3 Hi Emb (n = 36)	27.0 (6.4)	32.6 (8.1)	4.79 (8.36)	
4 Hi Emb-Narr (n = 39)	30.0 (8.8)	34.0 (10.3)		

### Engagement survey

The engagement survey was broken into two sections of interest: (1) increase in interest in the topic; and (2) total engagement in the task. These are reported as Bonferroni group contrasts.

#### Increased interest in topic of electric fields

For the question “I am now more interested in Electric Fields” participants answered on a 1 (strongly disagree) to 5 (strongly agree) scale. An ANOVA revealed significant group differences between the four Conditions, F (3,161) = 5.05, *p* = 0.002. The increase in interest nicely matched our predictions and the layered design of the content (SD in parentheses): S&T, M = 3.05 (0.91); Low Emb, M = 3.51 (0.82); High Emb, M = 3.65 (0.69), and High Emb-Narr, M = 3.69 (0.83). Bonferroni analyses on the group comparisons revealed a trend that the S&T group was somewhat less interested in the topic post-intervention than the Low Emb group (*p* = 0.065); statistically significant differences were seen comparing the Low Emb group with High Emb (*p* = 0.008), and also when comparing the Low Emb group with the high Emb-Narr group (*p* = 0.004). This last result shows that, on average, participants in the narrative condition reported that they were more interested in the content compared to participants in the other conditions.

#### Total engagement on “the activity”

See Table 5 for engagement descriptives. For a more stable score on engagement overall, total engagement rating scores were calculated by summing participants’ ratings to four Likert-style items on the engagement survey:

1. This activity was engaging;
2. This activity was boring (reverse coded);
3. I wanted to complete this activity;
4. Overall, I found this learning experience to be worth the effort.

**Table 5:** Engagement means and SDs

Condition	Total engagement rating *M* (*SD*)	Largest difference *M* (*SD*)	Effect size (Cohen’s *d*)
1 S&T (n = 39)	13.6 (3.3)	13.6 (3.3)	0.66
2 Lo Emb (n = 45)	14.6 (2.5)		
3 Hi Emb (n = 42)	15.9 (2.5)		
4 Hi Emb-Narr (n = 39)	15.6 (2.3)	15.6 (2.5)	

An ANOVA revealed that the three embodied conditions were found to be significantly more engaging and worth the effort, F (3, 161) = 6.28, *p* < 0.001. A Bonferonni analysis revealed that the difference between the S&T and the Low Emb conditions was not significant (*p* < 0.47); however, two further comparisons were significant, between S&T and High Emb (*p* < 0.001) and between S&T and High Emb-Narr (*p* < 0.005).

#### Do engagement and group interact to predict learning gains?

A regression was performed using the gains on the keyboard-based test as the dependent variable. The independent variables were group and engagement rating. This model with two predictors was significant, F (2, 163) = 4.72, *p* < 0.01. However, adding the interaction of group by engagement rating did not increase the model’s predictiveness; indeed, the adjusted R^2^ was reduced by 0.01. The models were run with both orthogonal contrast codes and simple linear codes. Engagement increases according to group placement and then appears to plateau with the two high embodied conditions. There is not a significant interaction between engagement and group; in this study, level of engagement did not moderate the effect of group for learning.

#### Correlations

Pearson *r* correlations were gathered on knowledge gains and the engagement and interest survey questions. Not surprisingly, engagement and interest correlated highly with each other, *r* = 0.73, *p* < 0.001. The content knowledge scores differed by type of test once again. Gain scores on the keyboard-based knowledge test were significantly correlated with interest (*r* = 0.29) and engagement (*r* = 0.31, all *p*s < 0.006); however, gains on the gesture-based *Wacom* test were not correlated at all with interest (*r* = 0.02) or engagement (also, *r* = 0.02). Partialling out the variance associated with experimental condition did not substantially alter significance levels between the *Wacom* test gains and interest, nor test gains and engagement (all *p*s > 0.40). Whatever drives the gains on the *Wacom* test may not be associated with interest or engagement as measured in this study.

## Discussion

This study holds implications for several fields including educational media design, knowledge assessment metrics, and embodiment in science. The research questions were designed to address three constructs important to science education: (1) what effect does level of embodiment and active gestures have on learning; (2) is there a narrative effect associated with science simulations (in a laboratory setting); and (3) what are the effects of test interfaces, specifically will differential learning gains will be seen on more embodied, gesture-based tests.

### Symbols and text versus the embodied conditions

The college-aged participants in this study learned more from the content when embodied simulations were included. When tested with a more traditional keyboard-driven multiple choice and short answer format, all participants in the embodied conditions (both low and high) demonstrated greater learning gains. Thus, the first research question regarding whether students learn more when new media science lessons are embodied has been answered.

### Gestural boost

Within the construct of embodiment, some lessons will be more embodied: how does learning compare in the passive (low) embodiment condition versus the active (high) embodiment condition. The two high embodied conditions came in at the fourth degree according to the taxonomy for embodiment in education (Johnson-Glenberg et al., 2016) and the low embodied condition came in at the second degree. The prediction had been that when participants were able to be active and control the screen content via gestures and motion capture, then those participants would experience a “gestural boost” in learning. Using the traditional keyboard-based test metric, a significant difference in learning was not observed between the low and high embodied groups. However, when assessed with the more embodied *Wacom* tablet measurement of knowledge, a significant difference in learning was observed. The two high embodied groups that used gestures to, for example, create vectors in a 3D space, performed better on the two-dimensional (2D) gesture-based *Wacom* assessment measure. This was not a given; the *Wacom* test mechanics of tapping and dragging the fingertip did not appear to be intuitive for many of the participants and required several practice trials at pre-test. Although, once participants were comfortable with the mechanics, they moved assuredly and did not need reminders at post-test.

The high embodied conditions also afforded more agency and were designed to include multiple instances of representational gestures. The use of gestures while learning may have lightened the cognitive load by “shifting information from the verbal memory store to a more visuospatial memory store” (Cook & Goldin-Meadow, 2006). Recent work also suggests a “sleeper effect” (Brooks & Goldin-Meadow, 2016) associated with congruent movements and gesture on the learning system, so that even if immediate gains are not seen after being shown movements, learners in that study were able to solve equations better when tested later. Goldin-Meadow et al. hypothesize that gesturing may sow the seeds for later learning (Brooks & Goldin-Meadow, 2016).

From a neurobiological perspective, a concept can be described as a network created by linked cell assemblies that process emotional, linguistic, and sensorimotor information (Hauk, Johnsrude, & Pulvermüller, 2004). Words and knowledge are represented in distributed networks with different topographies, including perisylvian areas and others involved in processing perception and action (Macedonia & von Kriegstein, 2012). Via Hebbian learning the shape and activation strengths of networks change over time, so that with more instances of exposure, and especially more multimodal exposure via action, concept learning may be strengthened. The result is a more robust network of traces. This can also be thought of as a knowledge structure. Learning science with meaningful gestures may have effects on retention and the decay rate of the knowledge and information, as has been seen in gesture-based language learning studies (Macedonia & von Kriegstein, 2012).

### Game narrative

We had predicted that both learning and engagement would be positively affected by learners being presented with a narrative storyline to cohere the multiple science simulations. We hypothesized that the narrative would help the learners to cohere the elements and maintain motivation to finish the lesson. The predicted gain in learning was not seen, scores were not significantly different when comparing the embodied condition without narrative to the embodied condition with narrative, even though participants spent an extra 7 min on the task in the narrative condition.

On the other hand, the highest interest scores for the topic were seen in the narrative condition. When comparing the High Embodied-Narrative group to the Symbols & Text group, the difference was significant (*p* < 0.004). This last result shows that, on average, participants in the narrative condition reported that they were more interested in the content compared with participants in the other conditions. This interest did not translate into “engagement” with the task as a whole as seen by the second survey question. The difference between the interest and engagement from the High Embodied version to the High Embodied-Narrative version was not significant. We recommend future studies should use engagement as a mediator of learning. Many theorists claim that adding game-like components will make the content more engaging and that higher engagement will lead to better learning, but large-scale RCT studies are difficult to find that demonstrate a causal or mediational role for engagement in educational videogames learning. We did not find a significant interaction of engagement by group in this study. For the keyboard test (only), those who were more engaged in the lesson did better, but performance was not also linked to, or moderated by group membership.

It is important that this narrative null result be reported so that it has a chance to be included in future meta-analyses. Speculatively, there may be several reasons why a narrative effect was not observed. First, people may be born “story makers.” That is, they may have simply induced their own narrative. Even though two of the embodied conditions did not know why they needed to stick balloons on a wall, or hit the dragon with lightning strikes, those participants were game to engage in those simulations without ever asking the experimenters “why” questions. Second, our storyline may not have been compelling to the college-age students, being in the role of an apprentice is somewhat low prestige. We would do well to heed advice from Marsh et al., that subtle character cues can have major effects on the research outcomes and in games, “characters are never socio-affect neutral” (Marsh et al., 2011). Third, and we hold to this one most strongly, narratives may be most effective when the content is delivered over several days because attention to learning may begin to flag only after the first novel exposure. This study is based on a single intervention exposure. If finishing lengthy, multi-session content is at the users’ discretion, then the power of the narrative might be observed. We have not seen published studies yet that compare learning gains on multi-day and user-controlled lessons with and without narrative wrappers. The Koenig studies cited earlier were one-shot lab experiments like this one. We know that entertainment videogames with rich storylines (and even very thin storylines like the Mario Bros. series) keep users returning. More research is needed on science education and the inclusion of narrative wrappers to understand if there is a true “value add” for the extra time and resources needed to create and comprehend quality narrative wrappers for multi-session content. In an experimental situation like this one, participants know they are going to get their credit regardless of performance or “grit”. Participants were going to finish no matter how dull the task because credit was the carrot and an experimenter was in the room the entire time. A narrative wrapper may boost motivation/engagement only when certain conditions are present, e.g. multiple homework sessions, duller simulations. We recommend the narrative wrapper research be done in situations prone to attrition and state unequivocally that this one lab experiment should *not* be interpreted as a paean to never waste funds on great narrative wrappers. Our take-away is quite specific, “If you design a *one-session science simulation lab experiment*, you may not see significant differences in learning associated with a narrative wrapper.”

### Test sensitivity

We had predicted that differential learning effects would be unearthed by an assessment that was more closely aligned with the method of encoding or learning. The two active, embodied conditions allowed participants to draw and move content on screen with large, congruent gestures. We predicted that the *Wacom* tablet which afforded participants the ability to swipe a screen with larger movements might reveal greater learning gains than the more traditional keyboard-based format. This prediction was supported and significantly greater knowledge gains were revealed for the two high embodied conditions on the *Wacom* test. It may also be the case that type of question is a factor, i.e. questions dealing with motion and forces at a distance may be better instantiated and assessed with the gesture-facilitated *Wacom* interface.

The *Wacom* was not a tool that any condition used during the intervention, so it can also be viewed as a transfer tool. This is the first time we have seen the large area tablet used in the science literature for such assessment purposes. We posit that such surfaces allow learners to show knowledge in a more embodied manner. The *Wacom* gestures are more congruent to the manner in which knowledge was learned in the final two high embodied conditions. In addition, by making participants actively “generate” the acceleration with their bodies, the test itself may have reinforced the learned concept, serving as a powerful multimodal prime, as well as being a more sensitive form of assessment.

It is intriguing that the *Wacom* test was able to distinguish a difference in learning between the lower degree embodied condition (second degree) and the higher degree embodied conditions (fourth degree). The two fourth degree lessons contained multiple instances of congruent gestures and far more sensorimotor activation (based on the Taxonomy for Embodiment in Education (Johnson-Glenberg et al., 2014a, 2014b, 2016)). Perhaps the increase in well-mapped sensorimotor activation created stronger memory traces and these facilitated better comprehension of the science content related to motion and forces at a distance. The comprehension that is gathered via more sensorimotor activation may be more easily tapped by measures that reactivate the sensorimotor system. Future studies should include non-gesture based questions on the tablet interface, i.e. multiple choice. In this manner, intra-indiviual correlations and increased validity for the measure can be garnered.

### Mixed/Virtual reality and gesture

In our theory, being active and using well-mapped gestures should facilitate deeper learning. When learners do the actions that are related to the concepts to be learned, they might not only be lightening the cognitive load, but strengthening the overall encoding signal by adding more modalities. Does this mean that more sensory input is always better?

The future of full sensory immersion in VR HMDs is intriguing for those in education. This lab is currently exploring how adding gesture via hand controls paired with HMD's will add to, or detract from, learning. VR and MR experiences can make the unseen be seen in a way that reading and 2D imagery cannot. By adding the gestural and haptic information to the educational experience, we would predict that a strengthening of the encoding signal will occur, provided the experience is properly designed and scaffolded.

Adding gesture to rich, highly immersive platforms may have further effects on learning. As an example of making the unseen be seen, when learners scuff on the carpet in the mixed reality lesson called *Scuff-n-Spark*, they are activating past memories of being shocked after walking on a carpeted surface. The experience includes digitized visuals, captured body movements, and well-mapped actionable content in a virtual world. Using many senses to transport learners back to mental models, or instances relatable to the real world, also enhances a feeling of presence or being there (Slater et al., 2010). This type of presence may serve as a prime for perceptual symbols to be activated or—using the language we prefer—it may prime the learner’s current knowledge structure. With the knowledge structure of a shock by static electricity primed and activated, it may be easier to integrate the concepts from the lesson, e.g. stripping carpet electrons with your feet makes the surface of your hand more negative. Showing the altering q_net_ on the virtual hand is an example of seeing the unseen. We hypothesize that when learners are visually and auditorally surrounded by the experience, more cognitive resources can be dedicated to adding new knowledge components to existing knowledge structures.

There are several directions for future studies. One might be researching how incrementally and systematically making a platform more immersive will affect learning and retention. We did not vary the platform in this study. For this study, we can say that using gesture-based controls had an effect on learning for the college-aged population recruited. We do not know how generalizable the results will be for younger students. We do not know if spatial skills moderate this type of embodied learning, that would be another interesting route for future studies.

### Design takeaways

We end with ten bullet points for strong design going forward. First, a recommendation is made that instructional designers who wish to create more embodied content deeply consider the affordances of their chosen technology; they should think through how gestures can be integrated with the technology and the content. They need to pilot those mechanics with each iteration. We chose to use a joint tracking sensor (the *Kinect*) for our most embodied fourth degree conditions because it afforded locomotion and gross motor gestures. However, if you know ahead of time that learners must be seated at a computer, there are still many creative uses for the mouse and tracking pad. Think about how the input device and user interface can be driven by gestures that are congruent to the content to be learned. For example, we wanted to build a simulation that would refute the misconception that a bob released from spinning would travel in a curved path (the impetus fallacy). A centripetal force simulation was created where the learner spins the bob on the screen by circling the mouse on a table and releases the virtual bob by lifting the index finger. This activity may recruit less sensorimotor activation than swinging a physical bob overhead (Johnson-Glenberg et al., 2016), but it is more active and embodied than viewing a video of the same. Design is always a trade-off.

Some concepts are clarified before the list is presented. Scaffolding, i.e. appropriately supporting the timing and amount of content, is crucial. In the series of electric field simulations, we started with a simple review on how to calculate the charge of an atom, then showed how a single test charge allows one to understand the electric field, then we allowed two charges to be free to move so that both q_1_ and q_2_ could interact. In the last and culminating simulation, participants explored the complexities of a lightning strike. Within each mini-lesson the sequence of content complexity and graphics in the user interface were scaffolded and added to. Creators should also design so that the learner can embrace failure. By allowing participants to construct and run models in the final two conditions, we allowed students to fail multiple times. Although in the Low Embodied condition pre-designed failures were shown, there may be something special about learning via your own failures. Failure in games is low stakes and critical for learning. Errors in a game-like setting provide valuable opportunities for learning when immediate feedback is provided. We use strong educational game design techniques (Gee & Shaffer, 2010) and always provided multiple, leveled trials with immediate feedback. Lastly, it cannot be action all the time, space must be built for refection as well.

The design tips are broken into creation of content and assessment categories:

*The Creation of the Content*

- Be embodied, be active
- Give a sense of agency
- Be gesturally congruent
- Scaffold components and complexity
- Encourage collaborative interaction
- Be error friendly
- Design in opportunities for reflection

*The Assessment of Learning*

- Be flexible, learning gains may show up in unexpected ways (maybe even only one month later)
- Embed in-game assessments
- If content is embodied, make assessment match

## Conclusions

With motion capture technology becoming more cost-effective and entering the education arena, it is important that embodied education experts discuss and design content in a more codified manner. As a field, we are in need of studies that explicate the most efficacious components in embodied science lessons. The study presented here assessed which variables predicted learning gains in a 1-h lesson. The three manipulated variables were: (1) level of embodiment; (2) level of active generativity; and (3) presence of story narrative. In addition, two types of tests were administered: (1) a traditional text-based physics test answered with a keyboard; and (2) a more gesture-based test using the *Wacom* large tablet. Results demonstrated that the three groups that included embodiment (both low and high) learned significantly more than the symbols and text group on the traditional keyboard post-test. When knowledge was assessed via the larger tablet format that facilitated gestures, the two active high embodied groups that learned with the *Kinect* sensor scored significantly higher on knowledge gains. This suggests that metrics should be developed that also assess knowledge that is gained in a more embodied manner. The metric should be valid and sensitive to the method of encoding. Engagement scores were significantly higher for the two active high embodied groups as well. The predicted differences in engagement and learning for the condition with the graphically rich story narrative were not supported. It may be the case that a narrative wrapper is not associated with learning benefits when short lessons are finished in one sitting, especially for lessons in a lab setting where the given reward is naturally going to be class credit. Narrative may yet be an appropriate mechanic for motivation when students are presented with longer lessons and they need to be interested enough to continue on their own. We recommend more research be done on the narrative effect in non-lab environments. We encourage science educators to consider how they can seek out, or create on their own, content that includes congruent gestures in new media.

### Additional file

Additional file 1: Declarative Knowledge Keyboard-based Test on Electric Field. (PDF 341 kb)

## Figures and Tables

**Fig. 9:**
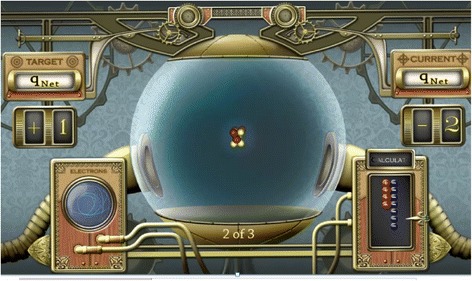
The Electron Counter

## Appendix 1

**Table 6:** Test questions and notes on the testers’ goals on the *Wacom* gesture-based test

Question		Notes
1	Imagine your fingertip is a charge that is free to move. Starting at the marker, simulate the movement of the charge as it moves to the top right with CONSTANT velocity.	Do they understand that the finger needs to move at a steady rate across the screen, i.e. “constant.”
2	Imagine your fingertip is a charge that is free to move. Starting at the marker, simulate the movement of the charge as it moves to the bottom right with CONSTANT velocity.	Give them practice in the opposite direction.
3	Imagine your fingertip is a NEGATIVE charge that is free to move. Starting at the marker, simulate the movement of the charge as it is positively accelerating to the top right corner.	Do they understand what it means to positively accelerate, i.e. move the finger faster towards the end of the swipe.
4	Imagine your fingertip is a NEGATIVE charge that is free to move. Starting at the marker, simulate the movement of the charge as it is negatively accelerating to the bottom left corner – that is the charge is slowing down.	Explicitly avoided testing simple vocabulary, thus we spell out “slow down;” will the finger mover slower at the end of the swipe?
5	Imagine your fingertip is a negative charge that is free to move. Starting at the marker, simulate how the charge will move in this scenario.	A positive red charge has been placed 4 units to the left of the start point. User should move towards the opposite ion showing positive acceleration towards the end.
6	Imagine your fingertip is a negative charge that is free to move. Starting at the marker, simulate the how the charge will move in this scenario.	A negative blue charge has been placed 4 units to the left of the start point. The user should move AWAY from the ion showing negative acceleration towards the end.
7 (and 8 appear on same screen)	Draw the force vector for the force being acted upon the red charge.	How would the blue electron be affected by the positive ion that is 2 units away? When scored this vector must be comparatively longer than the vector in answer 8.
8	Draw the force vector for the force being acted upon the red charge.	Here the electron is 6 units away, so the vector needs to be comparatively shorter than the one in answer 7 to receive full points.

## Appendix 2

### Choice of the topic and simulation details

**The topic: electric field**

The topic of the electric field was chosen because it can be a challenge to embody highly abstract content. The research in using concrete objects to teach abstract content is fairly established (Day, Motz, & Goldstone, 2015; Goldstone, Landy, & Son, 2008) in the cognitive and learning sciences. But, we do not yet know how very abstract concepts are learned when a mixture of digitized virtual objects are meshed with congruent gestures in a mixed reality situation. Generalization remains a thorny issue. Some research has shown that the more contextualized a training case, the more difficulty learners can have in “recognizing and applying its principles in new and dissimilar situations” (Day et al., 2015). Although participants often report being more “interested” in topics that are related to the real world and contextualized, transfer of knowledge has not been as strong with contextualized content compared to non-contextualized. Our working hypothesis is that embodied contextualizations of highly abstract and invisible (sub-atomic) content (i.e. rubbing electrons onto a virtual balloon) will lead to heightened interest in the content. The interest, and perhaps the addition of a gesture-based motor signal, may translate into an increase in comprehension.

STEM comprehension and learning depend on knowledge structure building. Novice science learners often possess unrelated and incorrect p-prims (diSessa, 1988); these are phenomenological primitives (simple abstractions that come from common experiences but are generally incorrectly applied in science). These pieces of knowledge do not hang together as a coherent scientific knowledge structure for many novice learners. A well-designed lesson should be able to prime learners to activate the correct knowledge, and then aid learners in placing the new pieces of knowledge into their existing knowledge structures. The lesson should encourage learners to construct coherent and verifiable conceptual models, manipulate those models to make accurate predictions, and then apply those models to make sense of new information. The models on the screen will eventually be internalized into working models in the learner’s memory. The lessons are constructivist in nature. Each lesson contains implicit scaffolding that regulates the amount of new knowledge learners are exposed to as they progress through the sequence and construct models. We were careful with the user interface and never added too many graphical components at once that might overwhelm the learner. The hints algorithm assured that they would not maintain incorrect p-prims. This science simulation advice is also touted by the PhET group that creates high quality science simulations https://phet.colorado.edu (Johnson-Glenberg et al., 2014a, 2014b).

**Simulation details**

(A) *Atom Builder – Low Embodied*. This simulation served to remind players of the structure of an atom and how charge is measured. Participants held the clicker and pushed the forward button to advance. They viewed three trial examples of electrons being added to the counting sphere, a glass sphere in the middle of the screen. In the center was a slowly spinning nucleus (with protons in red and neutrons in yellow). The goal was to match the target number for valence and an animation either added or deleted electrons to reach the target q_net_ (displayed in upper left corner).

Participants watched seven trials in total. They saw immediate feedback each time the calculate button was activated (correct or incorrect). The calculation action was shown with an arrow that moved down the calculate column. Where the arrow pointed was the place of the current sum and this would be displayed in the *Current* box. In Fig. 9, the current q_net_ is –2, but with one more tick down of the counting arrow it will reach –3 and “Incorrect” will be displayed because the *Target* value is +1. To reach the correct answer the animation will show four blue electrons being removed from the glass sphere in the middle.

For both the low and high versions of Atom Counter, there were seven trials total.

(B) *Atom Counter – High Embodied*. The *Kinect* sensor was always in front of the screen in all the conditions. Only in the two high embodied conditions was it automatically turned on and a red light would glow. The adding and deleting of electrons was controlled with the player’s highest hand, generally the right hand. When the wrist joint was read by the *Kinect* an open hand icon would appear on the screen so participants knew where their hand was. If the participant held his/her hand over the *Electrons* box (bottom left) or the central counting sphere and hit the advance button on the clicker, then the electrons would stop spinning and one electron would glow. The participant was then able to “grab” and move the glowing electron around on the screen. The electron would be released when the participant released the clicker button. Note: version 1 of the *Kinect* sensor did not have the “close hand” to grasp sensitivity, which version 2 has.

The choices were to either drag the electron from the *Electrons* box into the counting sphere, or to drag an electron out of the counting sphere. When an electron was dragged out of the sphere it could be released anywhere on the screen space and it would disappear. When participants were ready to submit an answer, they would click over the calculate box and the arrow would show up and begin to tally the electrons. The number under q_net_ changes with each tick down of the arrow in the *Calculate* box. The summation showed up in real time in the *Current* box. The first three examples always started with an incorrect match that needed to be corrected. As in the low embodied condition, the first three examples were animations showing the participants how the game worked; in conditions 3 and 4, items 4 to 7 were under the participant’s control to build. If the participant made three incorrect attempts the correct model would be constructed via an animation.

Simulation 2: Meter Made

To be learned: How free charges placed near pinned charges reveal the magnitude of the E field, includes dynamic equation

(A) *Meter Made – Low Embodied*. This simulation was designed to help learners understand that the strength of the E field can be assessed with a meter at one point in space. The meter has a charge of +1. The goal is to place the meter, currently filled with question marks in the second image in Fig. 5, so that it will match the *TARGET* E field (bottom left of screen), currently 1.000. The meter can be thought of as a free charge. This simulation is the first one to introduce the dynamic formula in the *Equation* box, upper left corner (second image in Fig. 5). The symbols change in size in real time to represent their magnitude based on what is happening on the screen.

In the middle of the screen is a pinned charged; it changes in valence and magnitude with each trial. The goal is to match the *Target* E field which reads 1.000. The participant watches the blue and red meter as it moves around the screen to the correct location where the E field is equal to 1.000. The system scores the submission of a meter reading with some leniency or “slop,” it allows the submission to be off by 0.05 for correct feedback. Participants watched animations of the meter placement for three practice trials, and five more trials including correct and incorrect first submission for a total of eight trials.

(B) *High Embodied – Meter Made*. This simulation also worked with a mixture of the *Kinect* reading the position of the highest wrist joint and the participant clicking the clicker to signal the submission of an answer. In the high embodied condition participants used their highest hand to control placement of the meter. When the participant was satisfied with the placement of the meter s/he would hit the forward button on the clicker held in the dominant hand. Immediate feedback was displayed. If the placement was incorrect three times in a row, the system will show the correct answer as an animation.

Participants could explore how the E field varied in all directions around the pinned charge of +2 in the center. The equation in the top left corner would change dynamically depending on distance (radius or r^2^).

Simulation 3: Vector Van Gogh

To be learned: Vectors in the E field reveal its magnitude and direction, included dynamic equation.

(A) *Vector Van Gogh – Low Embodied*. This simulation was designed to help participants understand the concept of vectors as possessing both magnitude (length of the arrow) and direction (the direction connotes attraction or repulsion). Participants are able to further explore how the strength of the E field can be assessed with pinned and free charges. See the third image in Fig. 5.

The participant would watch vectors being drawn from a circular “start point” with a dynamic measurement under the point. The simulation was nicely scaffolded for the participants, such that they first saw two vectors drawn in a gray shade in an animation, they then saw yellow vectors created on top of the gray shadowed ones, as if the vectors were being hand drawn. Two errors were always included in the sequence of eight trials.

(B) *Vector van Gogh – High Embodied*. The *Kinect* was used to track the highest hand. The clicker was held in the active highest hand. The goal of the high embodied version was for the participant to draw in the air the correct length and direction of the vector. When a participant would start to draw a vector the forward button was held down on the clicker and that button was released when the vector was finished. Similar to version A, this version also contained scaffolding. The first two trials were animations showing the answer. The next two trials used shadowed gray vectors to show the length and direction and the participants would trace over the gray vectors with yellow ones. In the final trials, participants would draw the vectors freehand. Immediate feedback was given after every submission. Per usual, participants were allowed three trials to get it right (0.05 match slop allowed). After the second attempt, a hint popped up to remind participants that the free charge that serves as the start point had a valence of +1. After a third error, the vector was animated for them.

Simulations 4a and 4b: Push Me Pull U and Mitey Electric Field Hockey

(A) and (B) *Push Me Pull U*. This served as an observational warm-up simulation for participants to explore vectors associated with two atoms. The dynamic equation in the upper left corner of now includes a numerator where q_1_ is multiplied by q_2_. The participants would click *Activate* at the top of the screen and observe how two charged particles would react in a contained space. Both particles would be released from a pinned situation at once and depending on their magnitude and valence, they would either head towards or away from each other. There were four examples. This simulation served as the introduction to the concept of how two free charges could affect one another so the relationship in the upper left corner now contains a q_1_ and q_2_. Only one version of this warm-up simulation was created it, thus, the same simulation immediately preceded to two different versions of Mitey Fields.

Simulation 4: Mitey Fields

To be learned: How charges work together to create a non-linear E field

(A) *Mitey Fields – Low Embodied Version*. Participants observed four simulations in this version. An animation showed how pinned charges could be placed on the screen from spheres below, see image 4 in Fig. 5. The pinned charge, for example the q = –5 charge, would be animated up from below and placed on the screen. Once *Activate* was hit the resultant E field would carry the blue creature called the “mite” back into a hole. The mite always had a charge of +1. In the first trial, an animation showed only one charge brought up from the spheres on the bottom of the screen (note in the bottom of the screenshot), the middle sphere holds a charge of q = –5). The second animation used two charges of differing valence. The third animation had gold bars in place that blocked the mite, but not the E field. Participants would observe, after two error placements of the pinned charges, how the mite could travel in a non-linear manner. The fourth animation involved the complexities of the mite curving into the end goal.

(B) *Mitey Fields – High Embodied*. In the high embodied version, the participants were able to use their highest hand and the clicker to grab charges from the spheres on the bottom of the screen and pin the charges anywhere on the screen. The mite is always charged with +1 so placing the –5 charge behind the mite will make it head straight into the hole. The participants worked through the same four examples described above; if they made three errors in placement in a row and the mite did not go into the hole, the system animated the correct answer. In the final example with gold bars, players deduced that charges could pass through the gold bars but the mite could not.

Simulation 5: Balloon Rub – Friction and Induction

To be learned: Charging via friction

(A) *Balloon Rub – Low Embodied*. This simulation addressed two important topics. The first topic was friction and it was demonstrated with the classic rubbing of a balloon on one’s hair. To try to mitigate race and gender issues by showing an avatar, a stylized artist’s mannequin was used to represent the body on screen. On screen, a yellow balloon was rubbed up and down the side of the avatar’s head to demonstrate how electrons can be stripped from hair (see fifth image in Fig. 5). As the yellow balloon picked up more electrons the balloon side touching the hair turned to blue, this simulated the balloon becoming charged with electrons from the hair. The right portion of the screenshot is labeled “Hyper Zoom Camera.” The black strands represent individual hairs and the blue particles are electrons with a charge of –1 each. As the mannequin animated rubbing the hair faster, more blue electrons accrued onto the yellow balloon.

The second topic of induction was introduced as an animation wherein the mannequin pushed the balloon towards the wall and the balloon then stuck to the wall. This is where the Hyper Zoom point of view was extremely helpful. The participant was able to see, subatomically, how the electrons on the balloon surface interacted with the electrons in the wall. In the Hyper Zoom shot the yellow balloon side is speckled with extra blue electrons, and on the right side (in the wall) the blue electrons are balanced in the neutral wall. The blue electrons spin close to their nuclei before the balloon comes towards the wall.

Figure 5 shows the state a few seconds later when the balloon is stuck to the wall. Via induction, the extra electrons on the balloon’ surface have pushed the electrons close to the surface of the wall a bit further into the wall. The balloon’s negative surface is now strongly attracted to the positive protons near the wall’s surface. The balloon will stay on the wall until the balloon and wall surfaces return to a balanced state.

(B) *Balloon Rub – High Embodied*. In the high embodied version, the *Kinect* sensor tracked the participant’s right arm movements. Specifically, at 60 HZ the sensor measured how often the participant’s right wrist joint moved up and down and used the ratio of that movement to map to the velocity of the mannequin moving the virtual balloon up and down. The participants faced the screen and sensor, and were instructed to pretend they were rubbing a balloon on their hair. The mannequin on screen mimicked the participant’s right arm movements, the controlling algorithm for the system was designed using the distance from the top of the participant’s head to the shoulder. Using the distance from the top of the mannequin’s head to its shoulder, we mapped the balloon position to the same ratio, thus when the participant’s hand was 10% from the top of the human head, the virtual, on-screen balloon was also 10% from the top of the mannequin’s head. The balloon locations were clamped at the top and bottom of the detected human head so the balloon would move on a vertical axis and never go below the shoulder or above the head.

The velocity of the participant’s balloon rub/arm movement was used to apply force to a physics simulation of hair strands that was created using Unity’s built-in physics engine. The velocity of rubbing determined the rate at which electrons were transferred from the hair to the balloon. The velocity of the human’s wrist joint movement drove the on-screen mannequin’s velocity as the yellow virtual balloon moved on screen and changed color. The faster that the participants rubbed the balloon, the more electrons they saw being stripped from the virtual hair in the Hyper Zoom view. When a set number of electrons gathered on the balloon surface, the scene switched from friction to induction.

For the second topic of induction, when the participant straightened out his/her right arm, the mannequin’s arm would also straighten out and move the virtual balloon towards the wall. With the Hyper Zoom camera view, participants could see that the closer the balloon moved towards the wall (the yellow section in the Hyper Zoom heads up display), the further away the blue electrons inside the wall moved. The graphics are the same as in the low embodied Version A simulation, except in this high embodied version B, participants had more agency and direct control over the mannequin.

When placing the balloon, the algorithm used the length of the user’s arm (length from hand to wrist, plus wrist to elbow, plus elbow to shoulder) as the maximum distance. In addition, some space (approximately the width of the balloon) was added to the balloon’s position so that it would activate a stick or unstick graphic just prior to participants completely extending their arms. With the completion of the right arm extension gesture, participants could run the mini-simulation multiple times, i.e. moving the balloon towards and away from the wall to understand the movement of electrons between the two surfaces. In version A, the induction sequence animation was shown two times. In version B, the high embodied condition, participants would enact the simulation a range of one to four times, with a mode of two times. Participants clicked over the *Done* button when ready to move on.

Simulation 6: Scuff-o-meter

To be learned: How friction can strip electrons from a surface and so that the potential difference between two charged objects becomes large enough to induce a spark

(A) *Low Embodied – Scuff-o-meter*. In the low embodied version the participants watched four animations of a spark occurring between the virtual hand on screen and the silver “glow globe” or spark candle on the right. The glow globe appeared with a different charge in each of the four trials. In the sixth image in Fig. 4, the glow charge is set to q = 10. In the low embodied animation version the hand on the left side of the screen would animate back and forth rapidly picking up electrons via friction (similar to the balloon simulation). The charge, on the hand increased with each scuff back and forth. The charge associated with the hand at the start state is on top of the hand and reads q = 0. On the bottom of the screen capture is the scuff-o-meter and as electrons are accrued blue dots begin to fill in the small circles. This is an example of representational fluency, using both numbers and blue electrons to show the increase. As the hand comes closer to the glow globe, the equation box on the left is dynamically tracking distance (r) in the denominator.

(B) *Scuff-o-Meter – High Embodied Version*. In the high embodied version, the *Kinect* was used to track the user’s highest hand, as well as the position of the two knee joints. First, participants were instructed to scuff, that is shuffle, their feet back and forth along a 2-m-long strip of the carpeted room. Participants could see how many electrons they accrued as they scuffed back and forth. They could see electrons accrue both on the virtual hand (via the q = label) and as the blue dot electrons accrued on the circles on bottom of the screen (the scuff-o-meter). When participants decided they had gathered enough electrons to create a spark, they brought their human hand, which was mapped to the virtual hand, towards the virtual glow globe. If enough electrons had been gathered (the numerator was high enough) and the distance was short enough (the denominator), then a large white spark filled the screen followed by the words “GOOD JOB.” There were four trials. This was probably the most embodied scenario as it involved some locomotion.

Simulation 7: Dragon Shockra!

To be learned: Charge separation and some of the conditions for lightning

(A) *Dragon Shockra – Low Embodied*. In the low embodied version, the participants were told that they would see a simulation where pieces of equipment would be “zapped” from a dragon (see equipment in Fig. 4), points would be awarded when pieces were knocked off. Participants should “notice the correct conditions” that preceded a lightning strike. To wit, the q_net_ in the cloud would need to be high enough and the dragon would need to be close enough for a lightning strike. The simulation showed the positive and negative charges in the cloud separating. The negative electrons would dynamically accrue in the bottom of the cloud and the charge at the bottom of the cloud was tallied as q_net_.

This was a “scrolling runner game.” The foreground would scroll to the right and the dragon would appear to fly to the left, towards the cloud. The dragon simulated quasi-random movements. Because the dragon had a charge of +1, the r, or distance, of the dragon to the cloud was an important variable that effected when the lightning strike would occur. Participants watched the three minute animation that resulted in the dragon being struck three times. Because the trees also carried valences of +1, they were sometimes struck as well, e.g. if the cloud was highly charged and it moved low on the screen it would strike the closest positively charged object and that was sometimes a tree. When trees were hit with lightning, they would smolder and no points were gained. The game was designed so participant would deduce the interaction between: (1) q_net_, as measured in the bottom of the cloud (the numerator of the equation); and (2) the distance of the dragon (the denominator) to the cloud.

(B) *Dragon Shockra – High Embodied*. As in the low embodied condition, the cloud location was constrained to move within the top left quadrant of the screen (counted as 100 units vertical from top left corner). The seventh image in Fig. 4 shows the cloud in the far bottom right position. In the high embodied version, the *Kinect* was used to track the participant’s highest wrist joint. Thus, the participant’s hand position controlled cloud location. Once the timer started the 3-min countdown, the dragon would automatically “fly” toward the left edge of the screen. The foregrounded fence and light poles could scroll to the right to give the illusion of the dragon flying.

The participants controlled how close the cloud could get to the dragon. The dragon’s flight path was perceived as “quasi-random” and depended on the user’s performance. The dragon had a pre-set pattern of 15 different X, Y positions where it could move to on the screen. Every game, the dragon would move in the same pattern; however, the rate at which it moved was driven by participant performance. For example, the screen was 356 units wide and 200 units high. The dragon was limited to an invisible box 150 units wide × 8 units high, the dragon box was tethered to the right half of the display screen and 35 units below the center. The timing for a dragon shift in position was based on the number of times the dragon had been struck by lightning. Strikes knocked off machine pieces and three strikes or hits would knock off three large machine pieces. Thus, if a player was very accurate at the onset of the game, the game quickly became a little more difficult. The time value was 0.66 s multiplied by the number of hits. A dragon that had not been hit would jump position on screen every 1.98 s, a dragon that had been hit once would jump every 1.32 s, and a dragon that had been hit twice would jump every 0.66 s. It was challenging for first-time players to know exactly where the dragon would be and that was the sensation we pilot-tested to create.

These movement constraints made it so that players should not simply position the cloud to always be low in the sky because if the cloud were charged and low, it would strike the closest positively charged object. That object would sometimes be a positive tree. As in the low embodied version A, when trees were hit with lightning they would smolder, no points were gained and the cloud reset to a neutral charge. The goal was to get the cloud close enough to the dragon to strike it and knock off pieces of equipment while not hitting trees. Only the participants in the fourth condition, with the narrative, understood these pieces of equipment were from the “vector machine.” The play mechanic was designed so that participants could use their knowledge of Coulomb’s law to be strategic. If players brought the cloud down too early they were penalized with tree strikes. The cloud would reset to zero q_net_ (thoroughly mixed positive and negative charges in the cloud) after each strike and it took a few seconds to build a negative charge back up, so it was important to not waste too many strikes within the 3-min time limit. If players knocked all three pieces of equipment off the dragon before the time limit, the game still continued for the full 3 min to equate for time on task between conditions. The dragon would continue to move at the 0.66-s rate until the clock ran down.

## Footnotes

1 While we often make reference to Coulomb’s law in this article, it should be noted that in the lesson the formula is often represented as a proportionality. The majority of participants were Psychology majors who may not have had much exposure to advanced physics (this has been our previous experience with this southwestern population). We did not include Coulomb’s constant (*k*) in the equation. We did not want to potentially confuse participants by also defining Coulomb’s *k*. They should focus on the concepts of a force carrying a charge, and the complexities of squared inverse proportions. Thus, the force was presented as F ∝ (q_1_ * q_2_)/r^2^.

2 In the instructional text it is stated, “A relationship exists among:

- ° the strength of the electromagnetic force between the two particles
- ° the net charge (q_net_) for each particle and
- ° the distance between the two particles

The relationship can be expressed mathematically:

$$ \mathrm{F}\propto \frac{\left({\mathrm{q}}_1\ast {\mathrm{q}}_2\right)}{{\mathrm{r}}^2} $$

q_1_ = net charge of particle 1 q_2_ = net charge of particle 2 r = distance between the two particles” Coulomb’s constant, *k* = 8.99 × 10^9^ N m^2^ C^−2^ is very small and not addressed. The concern was that for novice science students, it would be needlessly confusing.
