# Supplementary material for: Embodied science and mixed reality: How gesture and motion capture affect physics education
Source: Cogn Res Princ Implic. 2017 May 24;2:24. doi: 10.1186/s41235-017-0060-9 (PMC5442198; doi:10.1186/s41235-017-0060-9)
Supplement: Supplementary file 1 — Declarative Knowledge Keyboard-based Test on Electric Field. (PDF 341 kb) [file 41235_2017_60_MOESM1_ESM.pdf]

Additional file 1

Electric Fields Test v16a

Administered on Survey Gizmo

(One question appeared on screen at a time.)

Throughout the test, **ignore the effects of gravity. The term interaction refers to a push or a pull caused by a force.** Some of this may be new information for you but just try your best – you will learn some of it today.

1. Label the parts of the atom [shown below] and insert the charge.

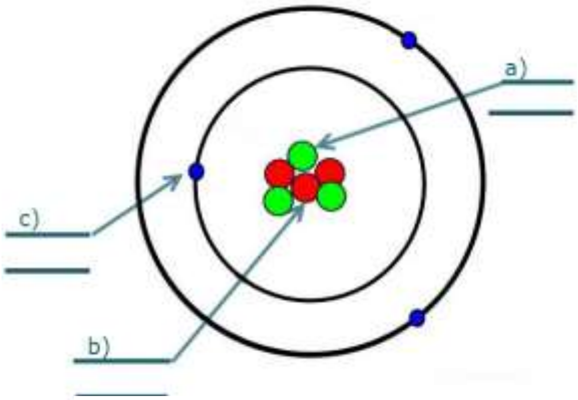

b)Proton

2. Identify the following atoms as being charged or uncharged. If charged, indicate whether they are charged positively or negatively. (n = neutron, p = proton, e = electron) What is net charge?

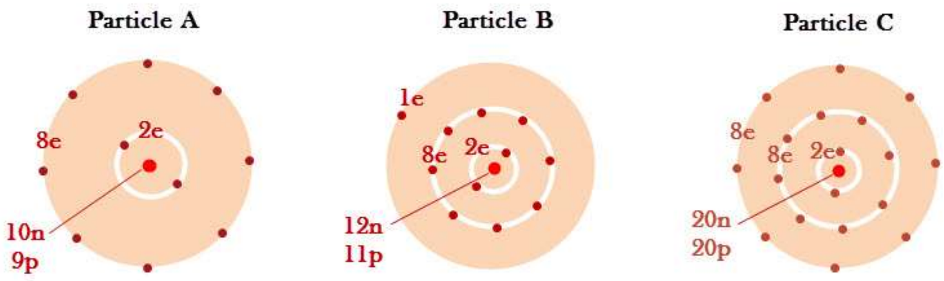

a. _________ b. _________ c. __________

3. The electrical interaction between charged particles located a distance from one another is carried by a ____________.

a) Field
b) Force
c) Charge
d) Voltage

4. A neutral plastic strip is rubbed with cotton and becomes positively charged. Why does this happen?
   a) The strip gained electrons from the cotton.
   b) The strip gained protons from the cotton.
   c) The strip lost protons to the cotton.
   d) The strip lost electrons to the cotton.

5. Charges that are similar ________________ each other,
6. while charges that are opposite____________ each other.

7. Two oppositely charged spheres are suspended from the ceiling, as shown below. In which direction will the spheres move when the strings are cut?

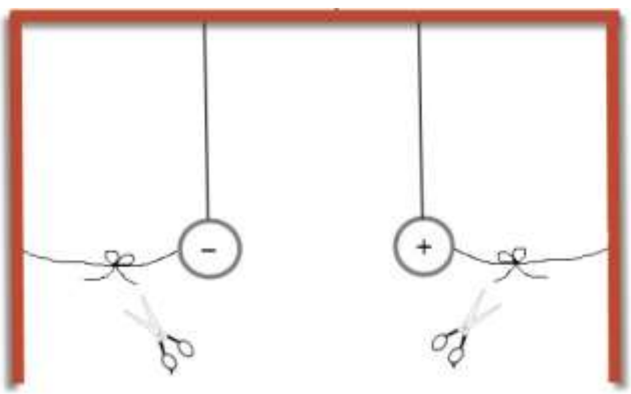

   a) ← →
   b) →
   c) ←
   d) → ←
   e) Will not move

8. Two positively charged spheres are suspended from the ceiling, as shown below. In which direction will the spheres move when the strings are cut?

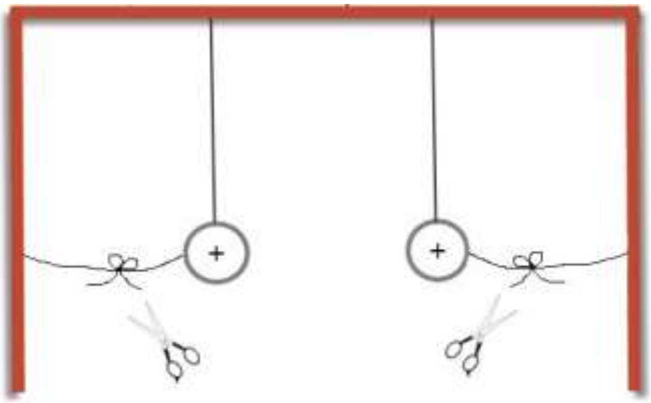

a) ← →
b) →
c) ←
d) → ←
e) Will not move

9. The electric field at a location in space is
   a) The sum of the fields of all charged particles when they are three units apart or less.
   b) The sum of the fields of all charged particles.
   c) A function of the charge of the particle at that location.
   d) The sum of the fields of all charged particles when they are one unit apart or less.

10. Consider an electron released in a large room. You "observe" that the electron begins moving upward, it is accelerating and the closer it gets to the ceiling the **more** it accelerates. This was most likely caused by
   a) A positively charged floor
   b) A negatively charged ceiling
   c) A positively charged ceiling
   d) A negatively charged floor

11. A negatively charged balloon is brought near a neutral sphere as shown below. As it approaches, the charges within the sphere will distribute themselves in a very specific manner. Which one of the diagrams below properly depicts the distribution of charges in the sphere?

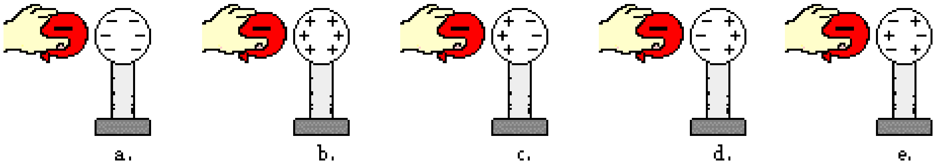

12. The symbol for charge is q.

Assume we have two fixed charges. Each has a net charge of +1, thus q_1 = +1 and q_2=+1. If q_1 is changed to +2. How does this change the net force between the charges?

a) It doubles.
b) It is cut in half.
c) It remains the same.
d) It is quadrupled.

12a) Explain your answer.

13. Assume we again have a net charge of +1, thus q_1 = +1 and q_2=+1. If q_1 is changed to -2. How does this change the net force between the charges?

a) It doubles.
b) It is cut in half.
c) It remains the same.
d) It is quadrupled.

13a) Explain your answer.

14. Using field (E), charge (q), and distance (r^2) or (r) , please write the equation that shows their relationship in the space below.

_______ = __________ / ___________

15. Two charges are located 10 cm from one another. If each charge is doubled in magnitude, the electric force they would exert on each other would __________.

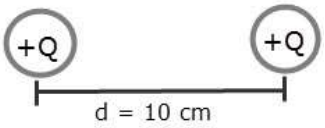

a) Double in magnitude
b) Halve in magnitude
c) Quadruple in magnitude X
d) Remain unchanged in both magnitude and direction

16. If the distance between two oppositely charged particles is cut in half then the force between the particles is

a) doubled
b) quadrupled
c) halved (cut down to 1/2)

d) quartered (cut down to 1/4)

17. If a point charge is fixed and a movable test charge is moved around it while maintaining a distance of 1 meter from the fixed charge, then ___________.
    a) only the magnitude of the electric field experienced by the test charge will change
    b) only the direction of the electric field experienced by the test charge will change
    c) both the magnitude and the direction of the electric field experienced by the test charge will change
    d) neither the magnitude nor the direction of the electric field experienced by the test charge will change

18. To the right is a picture of what was described in the previous question. What are two things you could alter on the test charge to make the direction of the vector change?

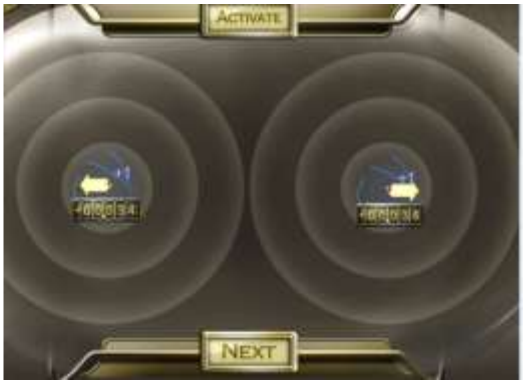

1)

2)

19. What is a spark?
    a) An electron travelling at the speed of light
    b) A particle of light emitted when an electron collides with a proton
    c) A particle of light emitted when electron collides with an air molecules between oppositely charged objects
    d) A particle of light emitted when an electron collides with a neutron

20. A spark can travel between your index finger and a doorknob when they come very close together. Assume that your finger is negatively charged and the doorknob is positively charged, so that excess negative charge transfers from your finger to the doorknob when the spark occurs. Why does this transfer happen?
    a) The strength of the electric field between your finger and the door knob decreases as the distance between your finger and the knob decreases

b) The excess negative charge on your finger decreases as the distance between your finger and the knob decreases
c) The strength of the electric field between your finger and the door knob increases as the distance between your finger and the knob decreases
d) The excess negative charge on your finger increases as the distance between your finger and the knob decreases

21. Imagine a cloud hovering above the desert. The bottom of the cloud is negatively charged. The surface of the earth is positively charged. If we place a) a positively and b) a negatively charged particle in the air between them. What will happen?
    a) the negative will _______________________________
    b) the positive will _______________________________
    c) In nature, we call this ___________________________
